# Complementary roles for mechanical and solvent-based recycling in low-carbon, circular polypropylene

**DOI:** 10.1073/pnas.2306902120

**Published:** 2023-11-07

**Authors:** Sarah L. Nordahl, Nawa R. Baral, Brett A. Helms, Corinne D. Scown

**Affiliations:** ^a^Energy Analysis and Environmental Impacts Division, Lawrence Berkeley National Laboratory, Berkeley, CA 94720; ^b^Department of Civil and Environmental Engineering, University of California, Berkeley, CA 94720; ^c^Joint BioEnergy Institute, Emeryville, CA 94608; ^d^Biological Systems and Engineering Division, Lawrence Berkeley National Laboratory, Berkeley, CA 94720; ^e^The Molecular Foundry, Lawrence Berkeley National Laboratory, Berkeley, CA 94720; ^f^Materials Sciences Division, Lawrence Berkeley National Laboratory, Berkeley, CA 94720; ^g^Chemical Sciences Division, Lawrence Berkeley National Laboratory, Berkeley, CA 94720; ^h^Energy and Biosciences Institute, University of California, Berkeley, CA 94720

**Keywords:** life-cycle assessment, plastics, greenhouse gases, recycling, circular economy

## Abstract

Plastic recycling presents a vexing challenge. Mechanical recycling offers substantial greenhouse gas emissions savings relative to virgin plastic production but suffers from degraded aesthetic and mechanical properties. Polypropylene, one of the most widely used and lowest-cost plastics, features methyl pendants along the polymer backbone, rendering it particularly susceptible to declining properties, performance, and aesthetics across a succession of mechanical recycles. Advanced processes, such as solvent-assisted recycling, promise near-virgin quality outputs at a greater energy and emissions footprint. Mechanical and advanced recycling are often presented as competing options, but real-world plastic waste streams are likely to require preprocessing regardless of whether they are routed to an advanced process. This study quantifies the life-cycle greenhouse gas implications of multiple recycling strategies and proposes a system in which mechanical and solvent-assisted recycling can be leveraged together to boost recycling rates and satisfy demand for a wider range of product applications. Polypropylene can be recovered from mixed-plastic bales produced at material recovery facilities and processed through mechanical recycling, with a varying fraction sent for further upgrading via solvent-assisted recycling to produce material approved for food packaging and other higher-quality applications. The resulting mechanically recycled rigid polypropylene reduces life-cycle greenhouse gas emissions by 80% relative to the same quantity of virgin material, while the upgraded higher-quality material achieves GHG savings of 30%.

## Significance

Polypropylene is a relatively low-cost polymer with useful material properties, making it one of the most widely produced plastics. Unfortunately, repeated mechanical recycling of polypropylene degrades its properties, performance, and aesthetics, so recycling infrastructure for polypropylene is underdeveloped and it often ends up in landfills. Solvent-assisted recycling processes like dissolution have emerged, offering near virgin-quality recycled polypropylene and the promise of greater circularity. To clarify the sustainability of circular polypropylene, we offer a detailed life-cycle evaluation of mechanical recycling, dissolution-based recycling, and virgin polypropylene production. We find that while dissolution-based recycling offers modest greenhouse gas savings relative to virgin polypropylene, it serves as an important upgrading step to broaden markets served by recycled polypropylene and displace demand for virgin resin.

Despite setting ambitious goals, most countries have struggled to reduce plastic waste accumulation, even in the face of growing evidence of its serious ecosystem, human health, and climate implications (1–3). These struggles have caused some to question the very premise that plastics recycling is a viable solution (4–6). In the United States, less than 6% of all plastics are recycled (7). Market forces, inadequate collection and sorting infrastructure, and an inability to produce virgin-quality recyclate have all played their roles in limiting recycling rates. Plastic recycling rates are higher in the European Union (averaging ~30%) due to stronger policies and higher tipping fees (8), but many of the same limitations stand in the way of further improvements (9). Some plastics are more easily recycled than others. Polyethylene terephthalate (PET) is arguably the easiest; it makes up just 10% of total US plastic production and yet 18% is collected for recycling (10–12). Polypropylene (PP), in contrast, makes up 14% of total polymer production and is recycled at a rate of less than 1% (7, 13). The low recycling rate for PP is driven by the same forces that hinder much of the plastic recycling industry: mechanical recycling results in an aesthetically and mechanically inferior product, making it difficult to compete with low-cost virgin material (14). The remaining question is whether, and how, greater circularity is achievable without driving up energy use and greenhouse gas (GHG) emissions.

Advanced recycling processes can create higher quality products, although often with higher energy use and emissions (15, 16). For example, pyrolyzing PP produces a diverse array of useful hydrocarbons, however, few of these get funneled back into virgin resin production and do so with low yields and high CO_2_ emissions (15, 17). Solvent-based processes have emerged as a strategy to more directly displace virgin plastic production with recycled material. Although solvent-assisted recycling processes can include either dissolution or depolymerization to monomers (also referred to as chemolysis), options for PP are currently limited to dissolution. Depolymerization to monomers (chemolysys) reverses a condensation reaction, which is not a viable option for addition polymers such as PP, PE, and polyvinyl chloride (18). This paper focuses on solvent-assisted dissolution of PP to produce polymer chains. This direct polymer-to-polymer recycling through dissolution and precipitation offers high yields and near-virgin quality with a reduced energy penalty relative to pyrolysis (15, 19, 20). We use process simulation and life-cycle assessment to show that, rather than treating mechanical and solvent-assisted PP recycling as competing options (21), enabling plastic circularity and driving down life-cycle GHG emissions will require both processes to be scaled in tandem. Maximizing mechanical recycling will minimize GHG emissions and produce low-grade recyclates for a market that has not yet been saturated. Developing solvent-assisted recycling processes as an optional upgrading step can provide higher-quality recyclates for a wider array of applications, including food packaging, while still achieving GHG reductions relative to virgin PP.

Our work indicates that a reframing of polymer recycling more broadly is necessary to develop realistic strategies for converting mixed plastic waste streams to recyclates that satisfy the diverse needs of the market, both for PP and potentially for other under-recycled polymers. By applying rigorous process simulation and life-cycle assessment informed by industry experts and real-world practices along the entire waste supply chain, our life-cycle energy and GHG emissions results provide the most industrially relevant insights to-date on how conventional and advanced recycling techniques can be leveraged to minimize GHG emissions and maximize waste diversion for one of the most commonly landfilled polymers on the market today.

## 1. Results

### 1.1. Impact of Sorting Constraints and Contamination.

Sorting constraints and realistic contamination levels are overlooked in much of the advanced recycling literature (15, 22). While the energy footprint of physical sorting processes is modest, melt filtration and other steps required to remove contaminants downstream can substantially impact energy use and yields. Hence, the design of recycling processes is dependent on sorting practices at material recovery facilities (MRFs). Most MRFs in the United States remove PET (#1) and high-density polyethylene (HDPE, #2) separately, then produce mixed plastic waste bales that include PP (#5) and other plastic waste, known as #3–7 bales. The composition of these bales varies considerably by individual MRF; for our analysis, we assume that PP (#5) makes up 59% of our input bale (*SI Appendix*, Fig. S1).

Upon arrival at recycling facilities, additional sorting is required to improve PP purity prior to dissolution or other advanced recycling processes. Solvent-based recycling is tolerant to contamination by other plastics, but increased plastic contamination in the incoming stream makes solvent selection and separation more challenging, so single-polymer feedstocks are preferable (19). Additives and dyes are also of minimal concern for solvent-based recyclers seeing as dissolution is capable of separating these impurities. The additional sorting or “preprocessing” necessary for PP dissolution includes the same steps that are used prior to mechanical PP recycling. This means that mechanical recyclers could preprocess a larger quantity of material to produce clean PP flakes and then choose, based on market conditions, what fraction to extrude on-site vs. export to solvent-assisted recycling facilities. Like mechanical recycling, dissolution-based recycling includes an extrusion step. However, at dissolution-based facilities, solvents are added to reduce shearing during this step and enable the eventual removal of shorter-chain polymers (23).

### 1.2. Life-Cycle Greenhouse Gas Impacts of PP Production and Recycling.

**Fig. 1.**
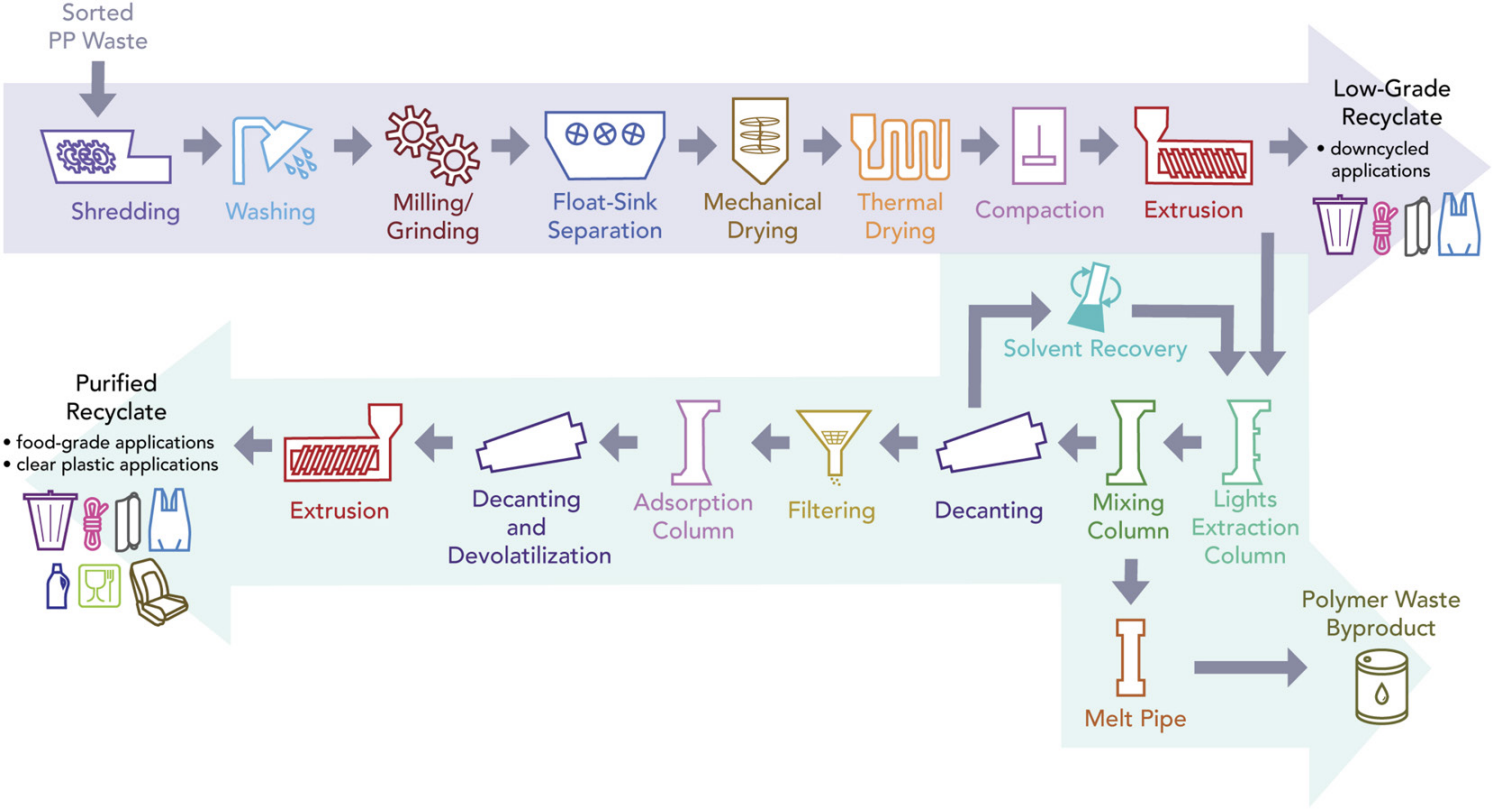
Polypropylene recycling with solvent-assisted upgrading. This flow diagram depicts both mechanical and solvent-assisted recycling. The processes highlighted by the purple arrow and ending with low-grade recyclate describe traditional mechanical recycling. The processes highlighted by the green arrows show how solvent-assisted upgrading produces purified recyclate from low-grade recyclate. The upgrading process also produces a waste polymer byproduct stream that can be used at petrochemical refineries. This diagram is adapted from information in academic literature and industry reports (17, 22, 26, 27).

The goal of this life-cycle assessment is to compare cradle-to-gate GHG emissions from dissolution-based recycling of rigid PP waste with conventional mechanical recycling and petroleum-derived virgin PP production in the United States. In all scenarios, the functional unit is defined as 1 tonne of PP resin produced, although, as shown in Fig. 1, recyclate quality varies by process. We use facility-scale industry data in the open literature to assemble mass and energy balances for conventional mechanical PP recycling and virgin PP production. Our model of the PP dissolution process is based on pilot-scale operations documented in industry reports (additional detail in the *Methods* and *SI Appendix*, Table S2), although our results are not specific to any individual site. The boundary of the analysis begins with raw material extraction in the case of virgin production and with plastic waste sorting for the recycling scenarios. Both recycling processes begin with mixed #3–7 bales (24, 25). Our analysis ends with the production of PP polymer resin that is ready for manufacturing, with the acknowledgement that the output from mechanical recycling cannot be used for all applications of virgin or solvent-assisted recycled PP. Further details on each recycling scenario are included in the *Methods* and virgin PP production is described in *SI Appendix*, section S1.

**Fig. 2.**
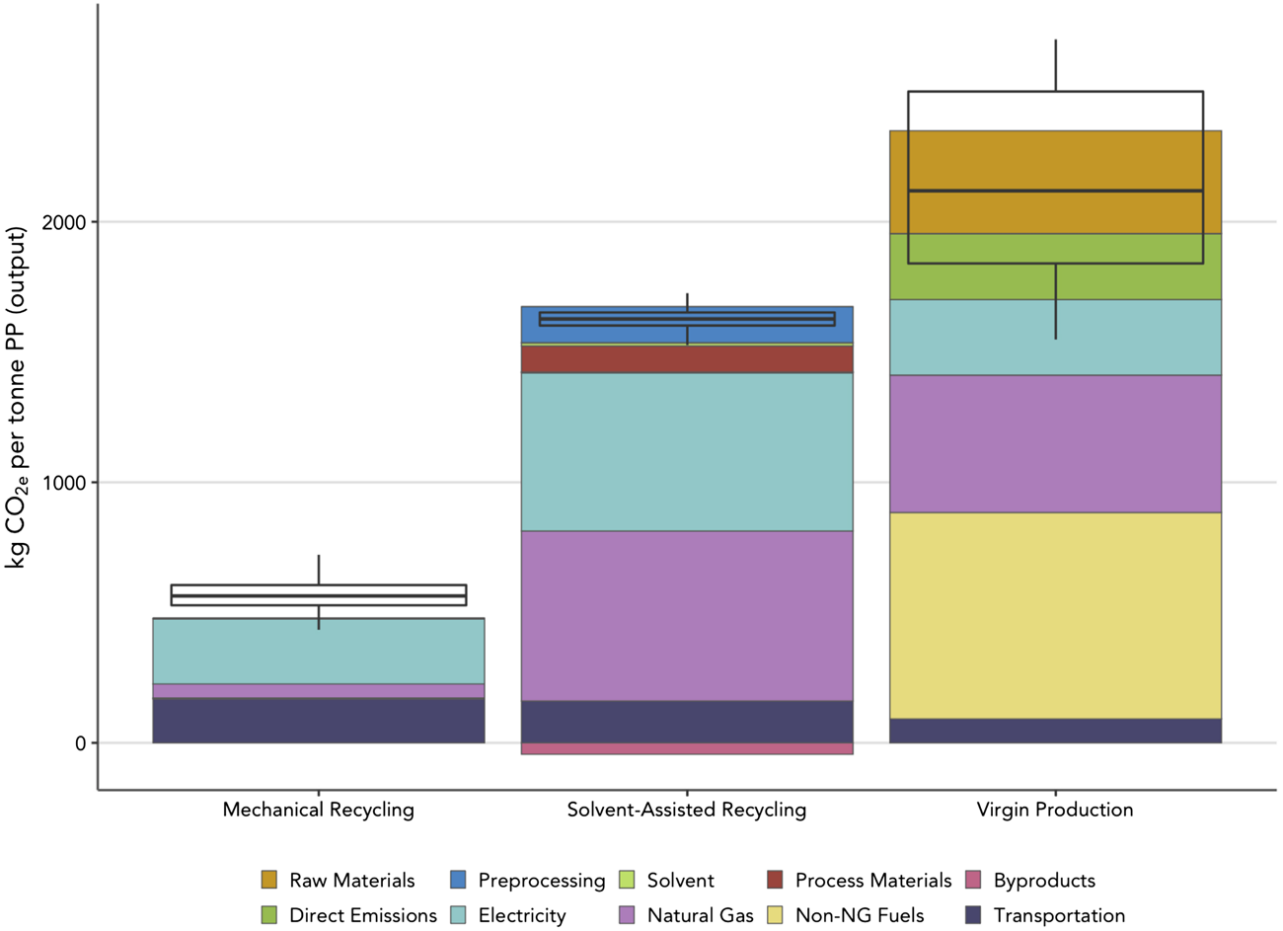
Life-cycle greenhouse gas impacts from virgin production and recycling. “Solvent-Assisted Recycling” refers to mechanical recycling with solvent-assisted upgrading. In the case of solvent-assisted recycling, “process” refers to dissolution and extrusion while “preprocessing” includes shredding, washing, grinding, float-sink separation and drying. Process energy consumption is broken down by electricity and thermal energy (from natural gas). GHG impacts from preprocessing energy consumption, both electrical and thermal, is included in the blue area labeled “Preprocessing” along with impacts from cleaning agents. The assumed grid mix is the US average for the recycling cases and the TRE NERC region for virgin production. The box and whisker plots for both recycling scenarios show the Monte Carlo results from a sensitivity analysis varying model parameters using probability distributions based on SuperPro results and literature values. Rather than a parallel sensitivity analysis, the box and whisker plot for virgin production depicts the distribution of estimated life-cycle GHG impacts for this mature process from literature.

Our results demonstrate that mechanical recycling is 70% less GHG-intensive than solvent-assisted recycling and 80% less GHG-intensive than virgin production on a per-tonne output basis (Fig. 2). However, mechanically recycled PP will be unsuitable for some applications and/or require blending with virgin material (*SI Appendix*, Fig. S2 and section S3). The solvent-assisted recycling scenario includes all upstream transportation, sorting, grinding, and extrusion impacts associated with mechanical recycling, with additional emissions associated with the dissolution process itself. Although the solvent-assisted process results in life-cycle GHG emissions nearly triple that of the mechanical recycling footprint, it still represents a 30% savings relative to virgin PP production. The box and whisker plots in Fig. 2 represent the Monte Carlo simulation results for the recycling scenarios based on parameter probability distributions with asymmetrical triangular distributions using mode, minimum, and maximum values from both literature and our process modeling in SuperPro (*SI Appendix*, Table S5). The probability distributions capture variation in pretreatment energy use, process energy use, process yields, and transportation requirements. The box and whisker plot for mechanical recycling is more offset from the bar graph than may be expected because the bar graph reflects average facility-scale operational data from Franklin Associates (28) while the probability distributions informing the Monte Carlo simulation are derived from our SuperPro results (*SI Appendix*, Table S5). Even after incorporating uncertainty, mechanical recycling remains substantially less emissions-intensive than solvent-assisted recycling. That said, the potential for process optimization in commercial-scale PP dissolution recycling is not captured by our analysis. Instead, we base our solvent-assisted recycling scenario on information and data reflecting recent, real-world operations still at pilot-scale to provide a conservative estimate of the associated GHG footprint. In contrast, mechanical recycling and virgin production are both mature processes, unlikely to change appreciably in the next decade. The range of life-cycle GHG emissions from virgin PP production is shown with the box-and-whisker plot for that scenario in Fig. 2.

The largest contributors to life-cycle GHG emissions for mechanical recycling are electricity consumption and transportation, which suggests that future grid decarbonization and electrification of freight trucks could increase the GHG benefits of recycling relative to virgin PP production. Approximately 45% of the life-cycle GHG emissions associated with solvent-assisted recycling are attributable to electricity consumption during both preprocessing and recycling. Another 40% of these emissions come from natural gas (including upstream and combustion emissions). Electricity consumption for virgin production is about 61% lower than that for solvent-assisted recycling. Impacts from virgin production are instead dominated by the consumption and combustion of petroleum-based fuels which are responsible for nearly 73% of life-cycle GHG emissions. While the results in Fig. 2 represent a snapshot of how each recycling process and virgin production compare given the current average US energy mix and incoming bale composition, it is important to note that both of these variables are likely to change by location and over time.

### 1.3. Greenhouse Gas Impact from Energy Consumption during Recycling.

**Fig. 3.**
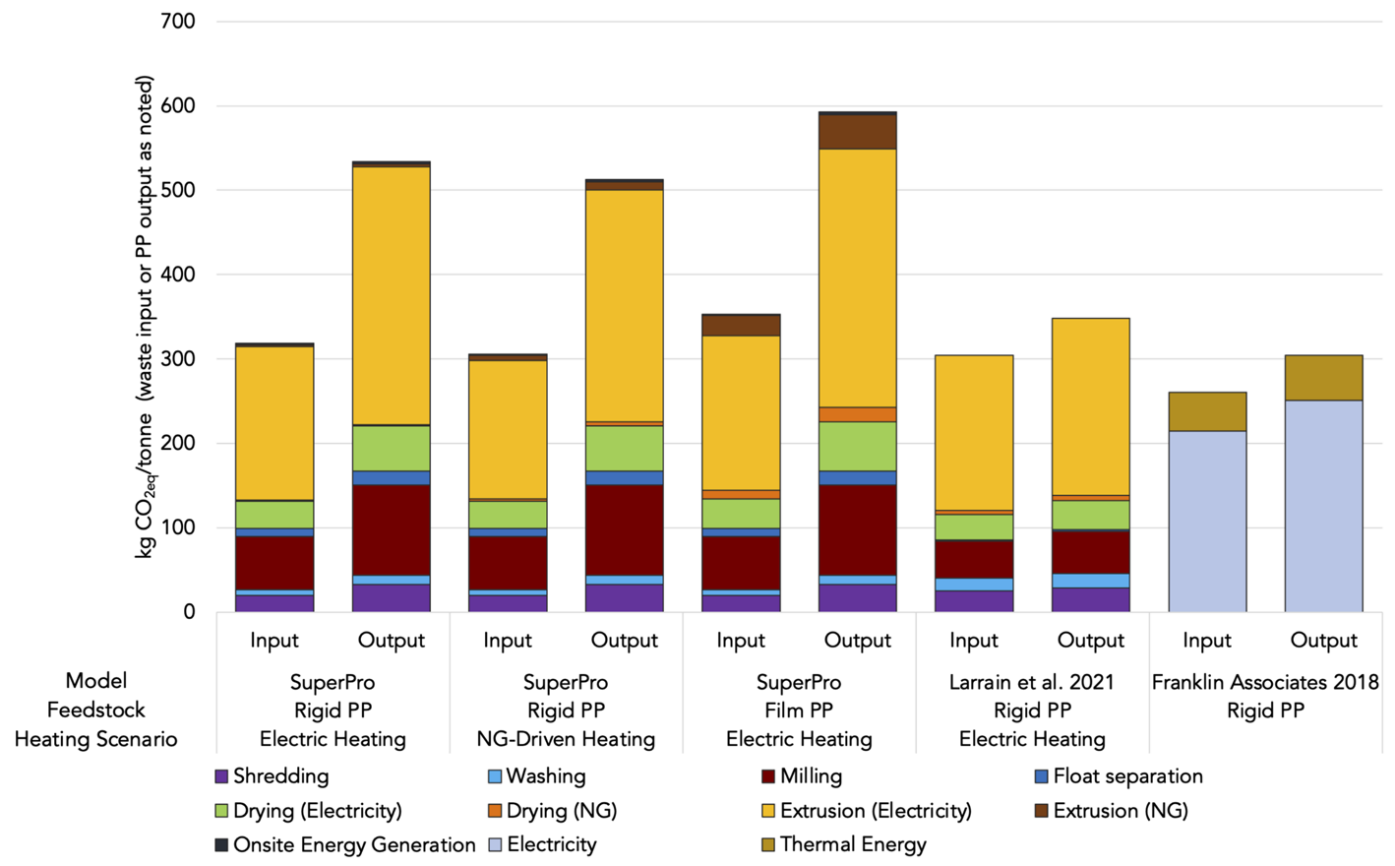
Energy-related greenhouse gas emissions by mechanical recycling unit process. The first eight bars (associated with four scenarios) show modeling results for facility-scale mechanical recycling operations broken down by unit process from SuperPro Designer and Larrain et al. (26). The last two bars depicted in this figure reflect the same operational data that was used for Fig. 2. There is insufficient information to break down this data by unit process so only GHG impact from total electrical and thermal energy consumption is reported.

The GHG impacts from PP production and recycling are primarily driven by energy-consuming processes and, particularly, for mechanical recycling, the breakdown of primary fuels vs. electricity can vary (Figs. 2 and 3). To better understand how and why the energy use and GHG emissions vary depending on equipment choices and the incoming waste’s form factor, we use process modeling to estimate impacts by unit process. Energy-related GHG impacts from operational data (shown in Fig. 2) are presented in Fig. 3 alongside modeling results for three mechanical recycling scenarios from SuperPro Designer; the corresponding energy consumption data are provided in *SI Appendix*, Fig. S4 and Table S1). The first and second scenarios depict mechanical recycling of rigid PP. The first scenario uses electric heating for extrusion while the second uses natural-gas driven heating. The third scenario reflects film PP recycling using electric heating. Because dissolution is a relatively new commercial process, we only have data for a single process configuration and thus do not conduct a similar unit-process-level analysis for solvent-assisted recycling. However, because dissolution requires similar preprocessing and extrusion steps, the results in Fig. 3 have implications for dissolution recycling as well.

The SuperPro modeling results for mechanical recycling shown in Fig. 3 highlight the importance of incoming waste stream purity and suggest that the GHG footprint of mechanical PP recycling in the United States may be higher than previously reported values from countries that recover PP separately. A prior study by Larrain et al. modeled mechanical recycling by unit process for a number of polymer types (26); their results for rigid PP are plotted alongside our results in Fig. 3. On a per-tonne input basis, our results are similar to those of Larrain et al. and to aggregated average operational data reported by Franklin Associates (2018), but on a per-tonne recyclate output basis, our results indicate higher energy needs and GHG emissions for mechanical recycling of rigid PP. For the most part, this can be explained by differences in input composition and therefore, final yield. While Larrain et al. modeled recycling of PP bottle bale, which is over 90% PP and available from sorting facilities in Europe, we model the recycling of a typical mixed #3–7 bale in the United States, which averages 59% PP. Franklin Associates (2018) does not report the incoming stream composition for their data, but their reported PP yield as a fraction of total incoming material (~85%) indicates that their incoming mix is likely more similar to that of Larrain (>90% PP) than a mixed #3–7 bale.

There are two important distinctions for mechanical recycling: film plastic vs. rigid and electric vs. natural gas-driven heating for extrusion. Recycling PP film requires more thermal energy relative to rigid PP because of the additional energy needed to dry the film plastic after washing and float-sink separation, given its higher surface area-to-volume ratio.

In the case of rigid PP recycling with electric heating, electricity makes up almost 98% of energy consumption while thermal energy from natural gas supplies the remaining 2%. Even when heat for extrusion is supplied by steam generated with natural gas (referred to as natural gas-driven heating), this thermal energy only contributes 8% of the total energy needs with the rest supplied by electricity. Operational data from Franklin Associates reflect the highest contribution from thermal energy to total energy-related GHG impact, close to 19% (Fig. 3). Our results from SuperPro modeling, along with those from Larrain et al., indicate even fewer GHG impacts from thermal energy. In all rigid PP scenarios, only 1 to 3% of energy-related GHG impacts are attributable to natural gas or other fuel consumption and in the case of film PP, up to 11%. The dominance of electricity in the overall energy requirements and subsequent emissions impacts for mechanical recycling suggests that the trend toward a decarbonized grid will reduce its GHG footprint over time.

Given the maturity of all the unit processes involved in mechanical recycling, any potential for future energy savings is likely to be small. Extrusion is the most energy intensive, contributing 56 to 60% to total energy-related GHG impacts. After extrusion, milling and drying are the next most energy-intensive processes. In the case of film PP, additional energy, particularly thermal energy for drying and extrusion, is required for mechanical recycling. Compared to the scenario with rigid PP and electric heating, drying and extruding film PP uses about 15% more electricity and almost 13 times as much energy from natural gas on a per tonne input basis.

### 1.4. Forecasting Polypropylene Production and Recycling Emission Factors.

**Fig. 4.**
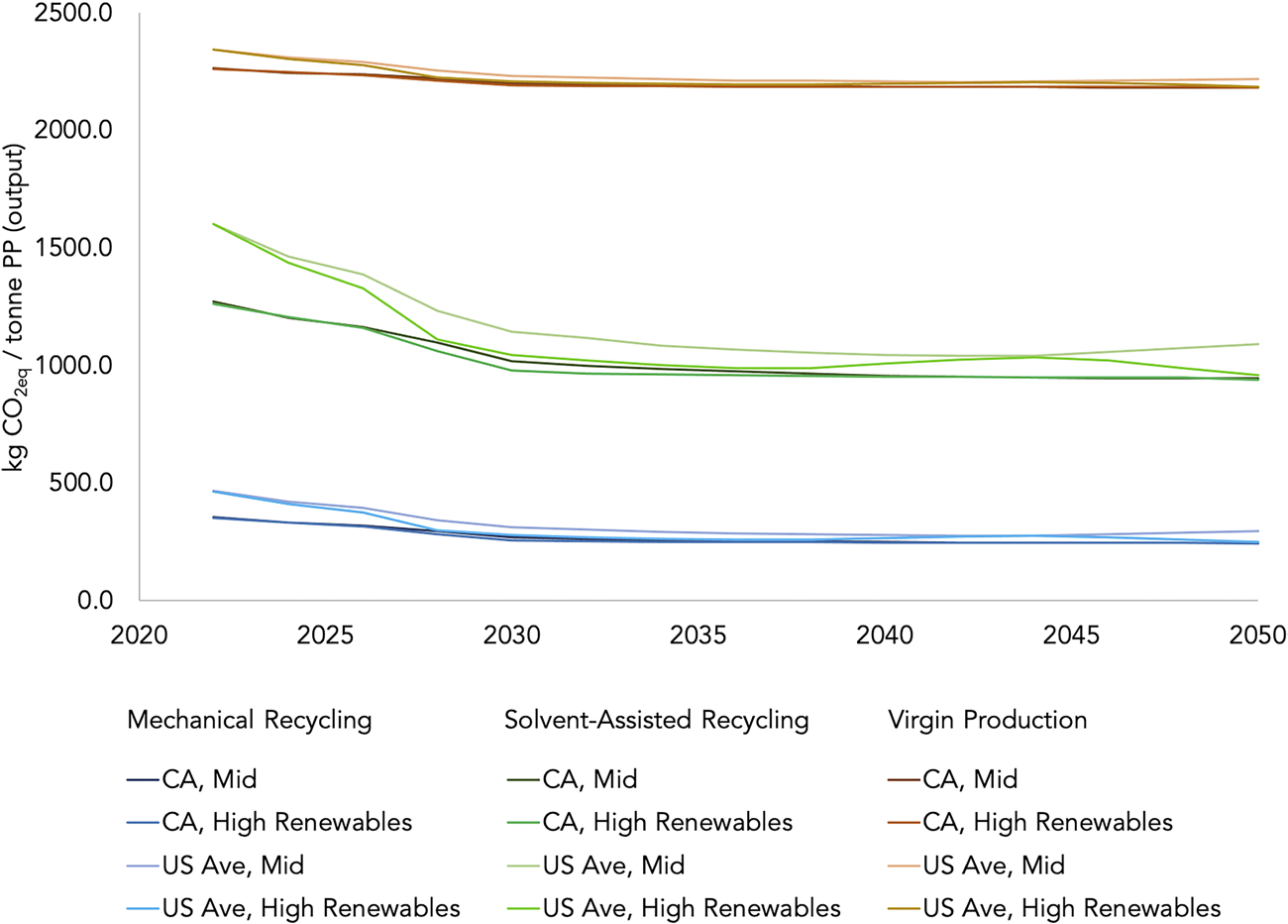
Emission factors forecast for polypropylene production and recycling. Sensitivity around electricity emission factor; using Cambium forecasts of grid carbon intensity for CA and the US average for a mid-case scenario (no nascent technology and current policies) and a high renewables case (low renewable energy cost, includes nascent technologies and decarbonization policy with goal of 95% GHG reduction by 2050). For this figure, we assume that electricity for each scenario is coming from the same source and grid mix (i.e., electricity source is not differentiated for virgin production as it was for Fig. 1). Upstream electricity demand is not updated in these forecasts but contributes less than 5% to the overall GHG footprint.

Because electricity is a large contributor to life-cycle GHG emissions in all scenarios, the assumption regarding grid mixes and electricity sources has a substantial impact on final results. The results for PP recycling presented in Fig. 2 reflect the US average grid mix for the recycling processes. We use the Texas Reliability Entity (TRE) North American Electric Reliability Corporation (NERC) region’s grid mix for virgin production because petrochemical production is concentrated in that region. Fig. 4 demonstrates how different and changing grid mixes impact the life-cycle GHG emissions from virgin production, mechanical recycling, and solvent-assisted recycling. We plot results using assumptions based on the average US grid mix and that of California (CA), a state whose grid mix is rapidly decarbonizing (29). Only the electricity directly consumed by production and recycling facilities is varied (upstream electricity use is not). Forecasts for future carbon intensities of electricity are based on two types of US electricity sector scenarios from the National Renewable Energy Laboratory’s (NREL’s) Cambium datasets: one standard “mid-case” with average costs assuming current policies and no nascent technology, and one “high renewables” case with low costs for renewable energy assuming nascent technology integration and 95%-decarbonization-by-2050 policy (30). As the carbon intensity of electricity decreases, the life-cycle GHG impact from PP recycling similarly decreases. In the plotted Cambium cases, GHG emissions from solvent-assisted recycling decrease by 26 to 40% in 2050 relative to 2022. Virgin production, on the other hand, is less electricity-intensive and the life-cycle GHG emissions from this scenario will not change considerably even if electricity is entirely decarbonized.

## 2. Discussion

### 2.1. Enabling Solvent-Assisted PP Recycling.

To facilitate the effective integration of solvent-assisted PP recycling in the broader system, new infrastructure plans should consider appropriate feedstock availability and market demand for recycled materials. While PP waste is generated by nearly every community and therefore should be obtainable in most places, high purity, sorted PP waste bales are not widely available from local waste sorting facilities and MRFs. The PP dissolution process does not require pure or contamination-free inputs (19), but input composition substantially affects the yield of recycled PP output, so sourcing high-purity feedstocks will be important for recyclers.

Since pretreatment for dissolution is essentially the same as mechanical recycling, mechanical recyclate is a potentially suitable feedstock for solvent-based reprocessing. It is important to note that PP material undergoes more quality loss during extrusion at a mechanical recycling facility than it does during solvent-assisted extrusion where solvents lower melt viscosity and reduce shearing (23). Additionally, extrusion is likely to occur on the front-end of any solvent-based process, regardless of previous extrusion, for size control and to enable a continuous process. To avoid needless extrusion and wasted energy, solvent-based recyclers should ideally source non-extruded excess material (flake) from mechanical recyclers. Beyond upgrading surplus recyclate from mechanical recyclers, solvent-based processes can serve as a recycling solution for hard-to-recycle materials (e.g., multilayer plastic packaging) that are not viable feedstocks for mechanical treatment (31).

### 2.2. Greenhouse Gas Footprint and Other Environmental Impacts.

Solvent-assisted recycling is already less GHG-intensive than virgin PP production, by about 30%, and is expected to become increasingly beneficial over time as the electricity generation mix continues to decarbonize. By 2050, dissolution-based PP recycling is expected to emit up to 40% less GHG emissions relative to virgin production based on expected average reductions in the carbon-intensity of the US grid. Mechanical recycling will continue to outperform solvent-assisted recycling on a GHG basis. Furthermore, the market for mechanically recycled PP is underdeveloped and far from reaching its technically feasible maximum, even after accounting for the range of PP product specifications. Increasing mechanical recycling will continue to yield GHG emission reductions. However, developing the infrastructure necessary to enable advanced processes such as solvent-assisted upgrading will be important for moving beyond a sole focus on downcycling.

This study focuses on the GHG emissions and energy use tied to PP recycling, but scaling up solvent-assisted PP recycling may also lead to other, non-GHG environmental benefits. Most obviously, scaling up any type of recycling process allows for more waste diversion from landfills. Apart from landfill diversion and GHG/energy benefits, which can be provided by mechanical recycling, solvent-assisted recycling, as a more circular technology, can offer virgin production offset credits across metrics. For instance, by enabling circularity and reducing virgin production, enzymatic recycling of PET can reduce smog formation, eutrophication, acidification, ecotoxicity, and human health impacts related to air quality (32). Using the same logic, we expect some additional sustainability benefits from solvent-assisted PP recycling and reduced virgin PP production beyond reducing GHG emissions and energy use. However, we do not have sufficient data to confidently analyze life-cycle emissions of non-GHG pollutants for the PP dissolution process. Additionally, several non-GHG environmental impacts (e.g., air quality and associated human health impacts) can be very location specific and this analysis is not tied to a particular site. Further research and emissions-related process data are required to confidently quantify other potential benefits.

## 3. Conclusions

As more companies and industry groups pledge to reduce reliance on virgin material, the gap between available recycled material and the required mechanical and aesthetic characteristics required will only become more obvious. Comparing advanced and mechanical recycling processes as competing options suggests a false choice; both are needed to process postconsumer plastics into recycled materials capable of meeting the wide range of quality materials demanded for modern manufacturing. This is particularly true for PP, where solvent-assisted recycling opens up the possibility of using recyclates in food contact materials and other applications that are still solely reliant on virgin material, while still reducing GHG emissions relative to virgin PP production. Furthermore, both mechanical and solvent-assisted recycling will become less carbon-intensive relative to virgin PP as the US electricity mix becomes cleaner and more reliant on renewable energy.

A key barrier to enabling lower-cost, lower-GHG recycling of all types is the level of contamination in plastic waste bales. Preprocessing steps, from milling to drying to extrusion all contribute to higher emissions and lower recycled plastic yields when incoming bales are highly contaminated. As novel recycling processes are developed and tested, incoming material must represent the full range of possible contaminants in real-world waste streams and future energy and mass balances should reflect realistic industry practices. To reduce the burden of additional preprocessing costs and energy penalties, countries seeking to increase recycling rates can invest in waste collection and sorting infrastructure needed to reduce contamination in waste plastic bales and enable the next generation of recycling facilities.

## 4. Methods

Across all recycling scenarios, we assume the initial waste input to the main recycling processes is a mixed #3–7 bale (*SI Appendix*, Fig. S1) from a MRF to represent realistic, current conditions. Because the energy footprint of MRFs is relatively small (about 5 to 8 kWh of electricity per tonne of waste throughput) (33), we assume the MRF energy and GHG emissions attributable to #3–7 bales, which only make up 3.7% of MRF throughput (10), are negligible in comparison to the more substantial energy and emissions associated with mechanical recycling and upgrading. *SI Appendix*, section S2 includes further discussion on plastic waste sorting. Curbside waste collection is not included in the scope of our analysis; we assume collection and transportation to a waste processing facility occurs regardless of whether and how PP is recycled. Because the impact of capital goods is uncertain and variable, we assume the impact to be negligible and exclude them from our analysis (34, 35).

### 4.1. Mechanical Recycling.

Mechanical recycling typically involves shredding, washing, milling or grinding, float-sink separation, drying, and extrusion (26). The process flow highlighted by the purple arrow in Fig. 1 describes conventional PP mechanical recycling. This process applies to most mechanical recycling of thermoplastics, although individual facilities may vary. Plastic waste entering a recycling facility is first shredded and washed to remove organic and water-soluble impurities. It is then milled for further size reduction before passing through a float-sink separation tank where polymer pieces are separated by density. The target polymer material is mechanically and thermally dried before extrusion, at which point material is heated and forced through a screw extruder. This is typically followed by a pelletizer or some other equipment to cut and shape the extruder output. Extrusion may include melt filtration to remove any remaining contaminants before the recyclate is ready for remanufacturing. There are material losses during mechanical recycling and residual waste polymer can be landfilled or incinerated for energy recovery. This will vary facility-to-facility, but for our analysis, we assume mechanical recyclers do not have on-site incineration and instead send residuals to landfilling. Because plastics take a long time to degrade in landfills, we assume that this has a negligible impact on GHG emissions. The output from traditional mechanical recycling is lower-grade material that cannot be used for all applications of virgin PP. Some studies account for this imperfect substitution between mechanically recycled and virgin PP by using a substitution factor, but we do not apply such a factor because these values are uncertain and product-specific (*SI Appendix*, Fig. S2). Detailed discussion on the limitations of mechanical recyclate and on the uncertainty of substitution factors is presented in *SI Appendix*, section S3.

In addition to using facility-level data on mechanical recycling from literature (28), we also model shredding, washing, milling, float-sink separation, drying, and extrusion in SuperPro Designer to understand the key drivers of energy use and trade-offs for different process configurations. Two studies were ultimately used as benchmarks for mechanical recycling: a report by Franklin and Associates and a study by Larrain et al. (2021) (26, 28). Franklin and Associates provide the average material energy balance data from three real-life PP reclaimer facilities but do not include breakdowns for energy consumption by unit process (28). Larrain et al. used a physical-based input–output process model to conduct a rigorous techno-economic assessment of mechanical recycling of PP along with polyethylene, polystyrene, and mixed polyolefins. They modeled the same unit processes although in slightly different configurations than what is included here (e.g., Larrain et al. model an additional milling step after thermal drying) and provide energy data by unit process (26). Across these studies, it is clear extrusion is the main driver of total energy consumption. To capture potential variations in energy inputs, we model two distinct cases for rigid PP extrusion: electric heating and natural gas (NG)-driven steam heating. Both are viable technology options, although electric heating is more common in the industry (36). Conversely, some unit operations consume such a small amount of energy that we have chosen to exclude them. Pelletization (cutting extruder output) is expected to have a negligible impact on the facility’s energy demand (<3% of extrusion impact) and the specifics depend on the desired form factor for remanufacturing, so it is excluded from the analysis (37). Similarly, we assume negligible energy impacts from compaction, which is not technically essential for recycling and depends on facility-specific configurations. If metal contamination is a concern, some facilities may also choose to include a magnet and/or eddy current separator on the front-end of their process. The addition of metal-removing equipment has an energy cost of less than 2 kWh per tonne input (33), less than any other unit process modeled for mechanical recycling (*SI Appendix*, Table S1). Because we assume low metal contamination (2%) in our initial recycling input, we do not include dedicated metals removal and instead assume that these contaminants are removed during float-sink separation.

### 4.2. Solvent-Assisted Upgrading.

Dissolution uses solvents to dissolve plastic waste and separate polymer chains from additives, dyes, and other impurities without involving the physical degradation of the original molecules. Selecting solvents depends on the target polymer being recycled and supercritical butane has proved to be an effective solvent for PP dissolution (15, 38). Another recent study models dissolution recycling of PP using xylene as a potential solvent and found the life-cycle GHG impact to be 2.2 kg of CO_2eq._ per kg of recyclate produced, almost 40% higher than our result (21). The process modeled for our study uses supercritical butane and involves a series of columns for extraction, mixing, filtering and adsorption, and then concludes with decanting and extrusion (Fig. 1) (27). After being dissolved, the PP solution is purified in the columns, where other contaminants are removed before being precipitated and extruded (15, 27, 38). The final output from dissolution is near-virgin quality recycled material. Publicly reported yields for PP recycling via dissolution vary based on original product forms but are as high as 99% for cups and containers in laboratory settings and as low as 32% for carpet fibers in pilot-scale facility testing (27, 39). Additionally, the dissolution process generates a secondary waste stream of polymer byproduct that can be used as a general hydrocarbon feedstock for the petrochemical industry (Fig. 1). Unlike mechanical recycling, solvent-based treatments of plastic materials, in isolation, do not noticeably impact the rheological, thermal, or mechanical properties of the polymer (19, 40). Because virgin production and solvent-based recycling produce PP of similar quality, these two production pathways can be directly compared (40, 41).

Important considerations for any advanced recycling process are whether contaminants are allowable and how material must be preprocessed. Early-stage tests are often done carefully chosen input materials and thus preprocessing requirements are minimal. Real-world facilities must be capable of handling a wide variety of contaminants that may include chlorinated compounds, metals, and flame retardants. Commercial-scale solvent-based recycling requires preprocessing of plastic waste beyond the basic sorting which occurs at an MRF (27, 42, 43). As depicted by Fig. 1, pretreatment to PP dissolution involves washing, grinding, float-sink separation, drying, and extrusion. In other words, solvent-based PP recycling (and likely other advanced recycling processes) occurs after the material is subjected to a series of preprocessing steps that closely resemble the entire mechanical recycling process. Industry interviews with plastic sorting and recycling facilities, along with publicly available reports and data, have confirmed that in commercial operations, extrusion is often used to filter out remaining impurities (through melt-filtration), enable continuous process flows, and to improve subsequent process efficiency (27, 44, 45). This is not only true in the case of solvent-based processes, but also for chemical recycling processes like pyrolysis (23, 44, 45). Systems already equipped to deal with solvents are particularly attractive hosts for extruders since the addition of solvents during extrusion can lower the melt viscosity, enabling better melt filtration and reducing material degradation (23). Because of this contrast between what preprocessing is required in a controlled laboratory setting and what is practical in commercial operations, some early-stage studies, including techno-economic analyses and life-cycle assessments, have likely underestimated the energy footprint of advanced recycling by omitting some or all of the preprocessing steps included in our study (41, 46). Rather than framing solvent-based recycling technologies as alternatives to mechanical recycling, they may be more accurately characterized as upgrading options that produce higher-value recycled material.

### 4.3. Life-Cycle Assessment.

To conduct the life-cycle assessment, we collected direct mass and energy flow data for each PP-producing/recycling process from process simulation models developed as part of this study and from literature sources. Those mass and energy flows then served as inputs to a physical units-based input–output life-cycle inventory model, Agile-Cradle-to-Grave (Agile-C2G) (47). The combined material and energy balance data for propylene production and conversion to polypropylene comes from literature (48). For the mechanical recycling scenario, we used data from literature that reflect average material and energy balance data from three real-life PP reclaimer facilities (28). For the solvent-assisted recycling scenario, we used SuperPro Designer to model mechanical pretreatment processes and used publicly available information supplemented with data from proprietary sources to model facility-scale PP dissolution (26, 27). Because this scenario includes polymer byproducts, which can be used as a hydrocarbon feedstock for other petrochemical processing, we employ system expansion in our analysis and conservatively assume crude oil production is offset by this byproduct stream based on an equivalent higher heating value. In practice, the use of the byproduct stream is uncertain and may end up being landfilled, providing no offset credits. However, changing this assumption does not have a significant impact on results as the credit provided by the byproduct stream is relatively small (Fig. 2). Film PP is excluded from the life-cycle GHG analysis because film plastics are not currently practical to recover at most MRFs and no data were available on solvent-assisted recycling of PP films. However, we did include a PP film scenario for mechanical recycling to provide a sense for how the energy balance differs relative to rigid PP. We used current and projected grid electricity carbon intensity factors from Cambium, which provides access to annual average emission factors for the NREL Standard Scenarios (30). We assume the US average grid mix for recycling facilities and the grid mix for the NERC region containing Texas, where petroleum refining and petrochemical production is concentrated, the Texas Reliability Entity (TRE) region. Other relevant emission factors and input–output data are assembled from literature sources, including peer-reviewed articles, GREET, and the Ecoinvent database (*SI Appendix*, Tables S3 and S4).

To capture recycling process variation, we established probability distributions for key parameters, including efficiencies and energy consumptions, based on previous literature and used these in a Monte Carlo analysis (*SI Appendix*, Table S5). The model was run for 10,000 trials drawing from these distributions to develop the box and whisker plots shown in the results for the recycling scenarios. We do not conduct a similar sensitivity analysis for virgin PP production because the technology is comparatively mature and the values provided here reflect the industry average. Instead, we reviewed recently published estimates for life-cycle GHG impacts of virgin production and presented the distribution of estimates as a box and whisker plot for the virgin PP production scenario (*SI Appendix*, Table S6).

## Supplementary Material

Appendix 01 (PDF)

## Data Availability

Some study data available Some of the underlying data we use is protected by an NDA. However, we have used public sources wherever possible to limit the number of datapoints that cannot be shared. All shareable study data are provided in the article and/or *SI Appendix*. Interested readers may contact Corinne D. Scown (cdscown@lbl.gov) for assistance in accessing data that cannot be made public.
