# Supplementary material for: Complementary roles for mechanical and solvent-based recycling in low-carbon, circular polypropylene
Source: Proc Natl Acad Sci U S A. 2023 Nov 7;120(46):e2306902120. doi: 10.1073/pnas.2306902120 (PMC10655212; doi:10.1073/pnas.2306902120)
Supplement: Supplementary file 1 — Appendix 01 (PDF) [file pnas.2306902120.sapp.pdf]

# Supplementary Information

# Complementary Roles for Mechanical and Solvent-Based Recycling in Low-Carbon, Circular Polypropylene

Sarah L. Nordahl[1,2], Nawa R. Baral[3,4], Brett A. Helms[3,5,6,7,*], and Corinne D. Scown[1,3,4,8*]

[1] Energy Analysis and Environmental Impacts Division, Lawrence Berkeley National Laboratory, 1 Cyclotron Road, Berkeley, CA 94720, USA
[2] Department of Civil and Environmental Engineering, University of California, Berkeley, Berkeley, CA 94720, USA
[3] Joint BioEnergy Institute, 5885 Hollis Street, Emeryville, CA 94608, USA
[4] Biological Systems and Engineering Division, Lawrence Berkeley National Laboratory, 1 Cyclotron Road, Berkeley, CA 94720 United States
[5] The Molecular Foundry, Lawrence Berkeley National Laboratory, Berkeley, CA 94720, USA.
[6] Materials Sciences Division, Lawrence Berkeley National Laboratory, Berkeley, CA 94720, USA.
[7] Chemical Sciences Division, Lawrence Berkeley National Laboratory, Berkeley, CA 94720, USA.
[8] Energy & Biosciences Institute, University of California, Berkeley, Berkeley, CA 94720, USA

[*]Co-corresponding authors: bahelms@lbl.gov, cdscown@lbl.gov

**Table of Contents**

1. Virgin PP Production
2. Sourcing PP Waste for Recycling Feedstocks
3. Mechanical Recycling
4. Solvent-Assisted Upgrading
5. Life-Cycle Assessment
6. Results: Tabulated Data

# 1. Virgin Polypropylene Production

Virgin polypropylene production involves two main processes: propylene monomer synthesis and conversion to polypropylene (PP). Propylene production happens alongside petroleum refining and the production of several other products, including other olefins like ethylene. In fact, propylene is often considered a byproduct of ethylene production (1). The main production process is thermal cracking or steam pyrolysis of fossil feedstocks (2). Propane is cracked to produce propylene and ethane is cracked to make ethylene. The "cracking" refers to the breaking of C-H bonds to allow for double bonds.

The process starts with feeding saturated hydrocarbons and steam to a hydrocracker where they are heated to ~1000ºC (2). In the case of olefin production, lighter phase feedstocks like LPG or light naphtha are preferable (1). To stop the reaction after sufficient heating, the cracked products are cooled with heat exchangers generating high pressure steam. The gas stream goes through a centrifugal compressor to remove fuel oil and then undergoes hydrogen sulfide removal. Lastly, fractional distillation separates the reaction products.

Propylene is converted into PP via free-radical polymerization usually with Ziegler-Natta (Z-N) or metallocene catalysts (1, 2). There are multiple processes used by industry to make polypropylene including gas-phase polymerization and solution or liquid-phase polymerization (2). In gas-phase polymerization, propylene vapor is mixed with the catalyst in a fluidized bed reactor (Figure 1) (2, 3). The reactor is typically kept at 80-90ºC with a pressure of 90-25 atm. Any gaseous propylene that does not react is added back to the feed stream. The result of the reaction is solid PP which is then dried and pelletized. Liquid-phase polymerization is conceptually similar except the feedstock propylene is liquid and the reactor is tubular.

We use mass and energy flow data for PP production, inclusive of both propylene synthesis and conversion to polymer, from a 2011 ACC (American Chemistry Council) report for analysis (2).

# 2. Sourcing PP Waste for Recycling Feedstocks

*Plastic Waste Sorting*

Our analysis begins with plastic waste sorting at an MRF. The efficacy and associated benefits of most recycling processes are highly dependent on feedstock composition and require waste materials to be sorted for reclamation (4). Initial sorting occurs at MRFs, where recyclable waste is separated by material type (e.g., plastics, fibers, metals, glass). Generally, MRFs also separate plastics by polymer type using optical sorters and near infrared (NIR) technology. Most MRFs in the U.S. today primarily target and bale PET and high-density polyethylene (HDPE), with a particular focus on bottles, during sorting (5, 6). Other polymers, including PP, can also be targeted for selective recovery using the same NIR technology. However, PP is more commonly baled with other non-PET, non-HDPE plastics in mixed bales. These mixed bales are typically called #3-7 bales referring to the resin identification codes for polyvinyl chloride (#3),

low-density polyethylene (#4), PP (#5), polystyrene (#6), and other plastics (#7). While #1 and #2 plastics, PET and HDPE respectively, are typically targeted for separate recovery, sorting efficiencies at MRFs are imperfect and some of these materials may end up in Mixed #3-7 bales. Mixed #3–7 bales include rigid product forms but exclude film plastics, which are harder to separate and recycle.

To represent current practices across most of the U.S., we assume the initial input to any PP recycling process is a mixed #3–7 bale (Figure S1) rather than a PP-specific waste bale from an MRF. Non-target (non-PP) materials are then separated out by float-sink separation and routed for disposal. If a wider variety of polymer types, including PP, become attractive candidates for recycling, MRFs may add new "lines" to recover these materials separately. Alternatively, so-called secondary MRFs may be constructed to take in #3–7 bales and further separate the material (7). Neither of these developments will dramatically impact the energy footprint of recycling. The energy footprint of sorting at MRFs (4.7–7.8 kWh of electricity per tonne of waste throughput) is small compared to the thermal and electrical energy needed during mechanical or advanced recycling processes (4). Because only 3.7% of current MRF throughput are #3–7 plastics (8), we assume the MRF energy and GHG impacts that can be allocated to #3–7 bales are negligible.

*Feedstock Type*

The assumed input for all of our recycling scenarios is a mixed #3-7 bale, the most typical bale containing PP available from MRFs. We initially assumed the bale composition reported by APR (The Association of Plastic Recyclers) in a 2017 presentation (9), which was derived from a 2015 APR report which goes into further detail on MRF bale compositions (10). This included an "Other" category of 11% but instead of having unknowns, we assumed the maximum allowable contamination of paper (2%), metals (assumed to be aluminum) (2%), and liquids (assumed to be water) (1%) as given by the APR's model bale specifications (11). The remaining unknown component (a total of 6%) was distributed evenly to PET, HDPE and PP. The final composition used for this study is presented in Figure S1 below. In reality, bales will vary considerably from MRF to MRF. Furthermore, sorting operations and subsequently, the composition of outgoing MRF bales, are constantly evolving with changing market conditions; as the price of different materials and polymers change, MRFs modify operational configurations and bring in new equipment to recover valuable, salable material.

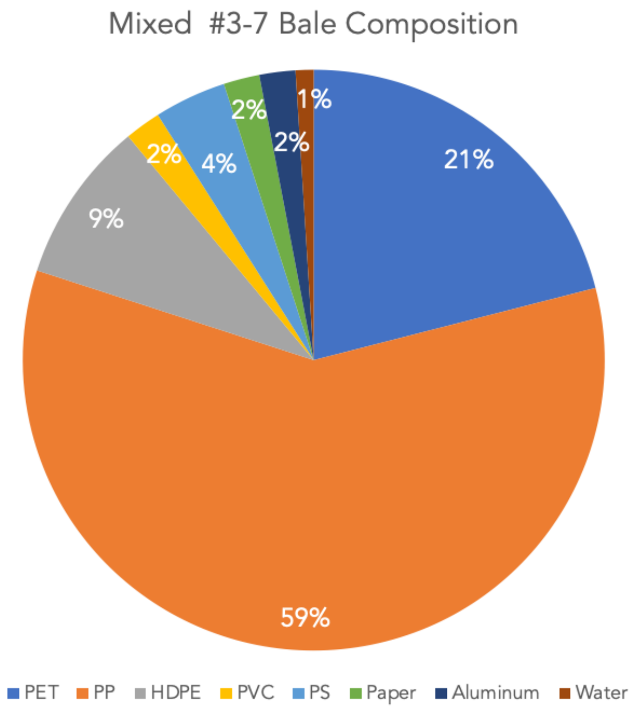


**Figure S1. Mixed #3-7 Bale Composition**
Adapted from 2015 APR report (9)

*Feedstock Transportation*

We assume that on average, transportation of material from site of sorting/separation (i.e. MRFs) to PP recyclers (mechanical or otherwise) includes 0.66 tonne-km of truck transport and 0.25 tonne-km of rail transport per kg of incoming material to recyclers (12). We assume flatbed trucking for truck transport.

## 3. Mechanical Recycling

*Limitations of mechanical recyclate*

PP can only undergo traditional mechanical recycling a few times before irrevocably degrading to unusable materials in circular manufacturing. PP has methyl groups on every other carbon atom, making it prone to chain scission during mechanical grinding and high shear rates during melt processing (13). In contrast, polyethylene (PE), the most common plastic, can withstand over 30 recycling cycles (13, 14). Repeated mechanical recycling of PP results in reduced molecular weight, increased crystallinity, reduced impact resistance, and increased opacity (15–19). Because of this physical degradation and difficult-to-separate impurities in plastic waste streams, the material produced from mechanical recycling is usually of lower quality than virgin resin (20). Mechanically recycled PP has inferior physicochemical properties than virgin PP. For most applications, PP recyclate must be blended with virgin polymer to ensure sufficient material performance (15, 21–23). For some uses including automotive applications and food-safe packaging, which have stricter thermal, chemical, and mechanical property requirements for safety reasons, PP recyclate can be deemed entirely unsuitable (20, 22, 24, 25).

*Substitution factors for mechanical recyclate*

Some studies account for the imperfect substitution between mechanically recycled and virgin PP by using a substitution factor (Figure S2). For example, 1 kg of mechanically recycled PP can be treated as functionally equivalent to 0.7–1.0 kg of virgin PP (26–32). Some studies may use terms like “substitution factor” or “substitution ratio” in reference to technical recovery efficiency of a recycling process (e.g., mass maintained versus lost during recycling process) (27, 33, 34), market substitution factor (e.g., additional material required to make a given product with lower-grade recyclates versus virgin-grade polymer) (26, 27, 31, 33, 34), value-corrected market substitution factor or displacement rate (e.g., price-based ratio describing market uptake of recyclate) (30, 34, 35), and blending limits (e.g., when blending with virgin material, the maximum allowable recycled content to avoid excessive quality loss) (23) (Figure S2). For the purposes of conducting a life-cycle assessment, clearly defining an appropriate functional unit (i.e., virgin material displaced per unit of waste plastic versus per unit of recyclate) is critical.

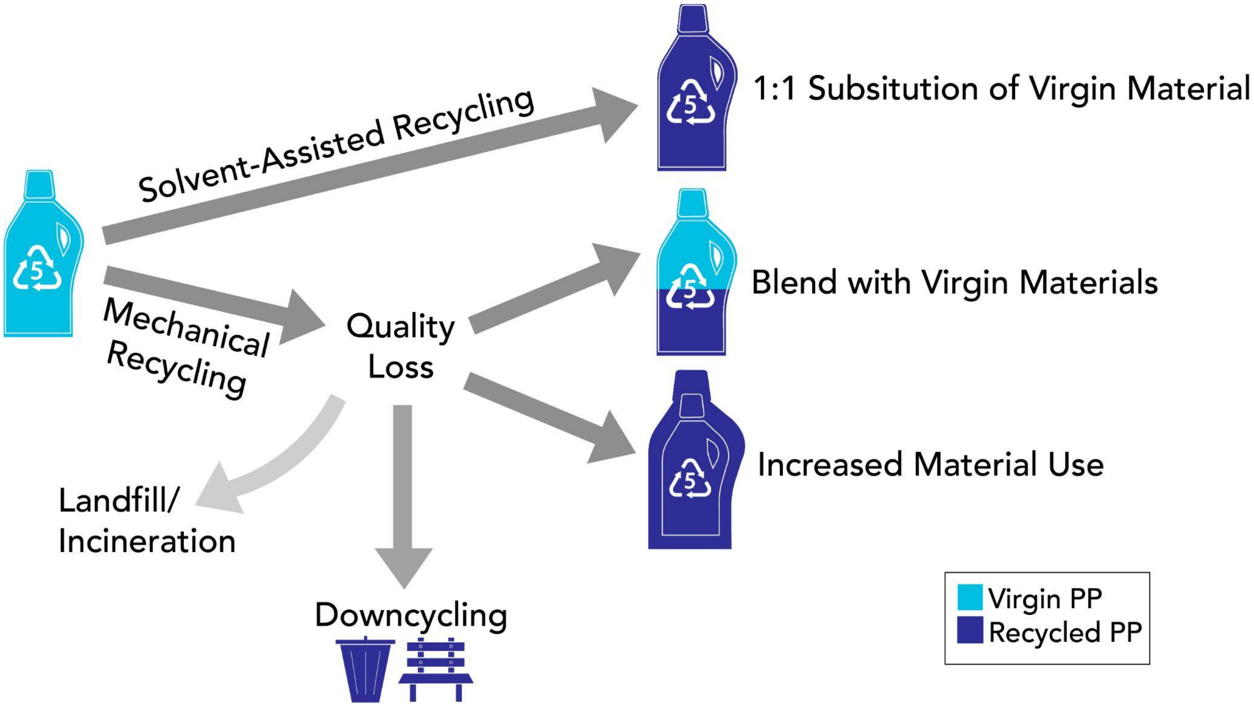


**Figure S2. Substituting Virgin PP with Recycled PP**

This figure depicts how recycled material can substitute virgin material use and the limits to perfect substitution for mechanical recyclates. Note that both recycling processes have mass losses which are not depicted in this figure; only loss to quality is included.

This study does not apply a substitution factor because these values are uncertain and product-specific. There are no currently available, robust estimates for how much virgin resin production has actually been offset by recyclates (36). While technical process efficiencies are well understood and can be measured directly for a particular recycling method, market substitution factors and blending limits are under-reported and variable depending on application and product type (37). Furthermore, the amount of recycled PP that the market could absorb is likely still much greater than the quantity available today. Until the industry-wide capacity for blending recycled PP has been reached for all product and application types, recycled PP can offset virgin material use on a 1:1 basis unless increased material use is also required (Figure S2).

*Process Modeling*

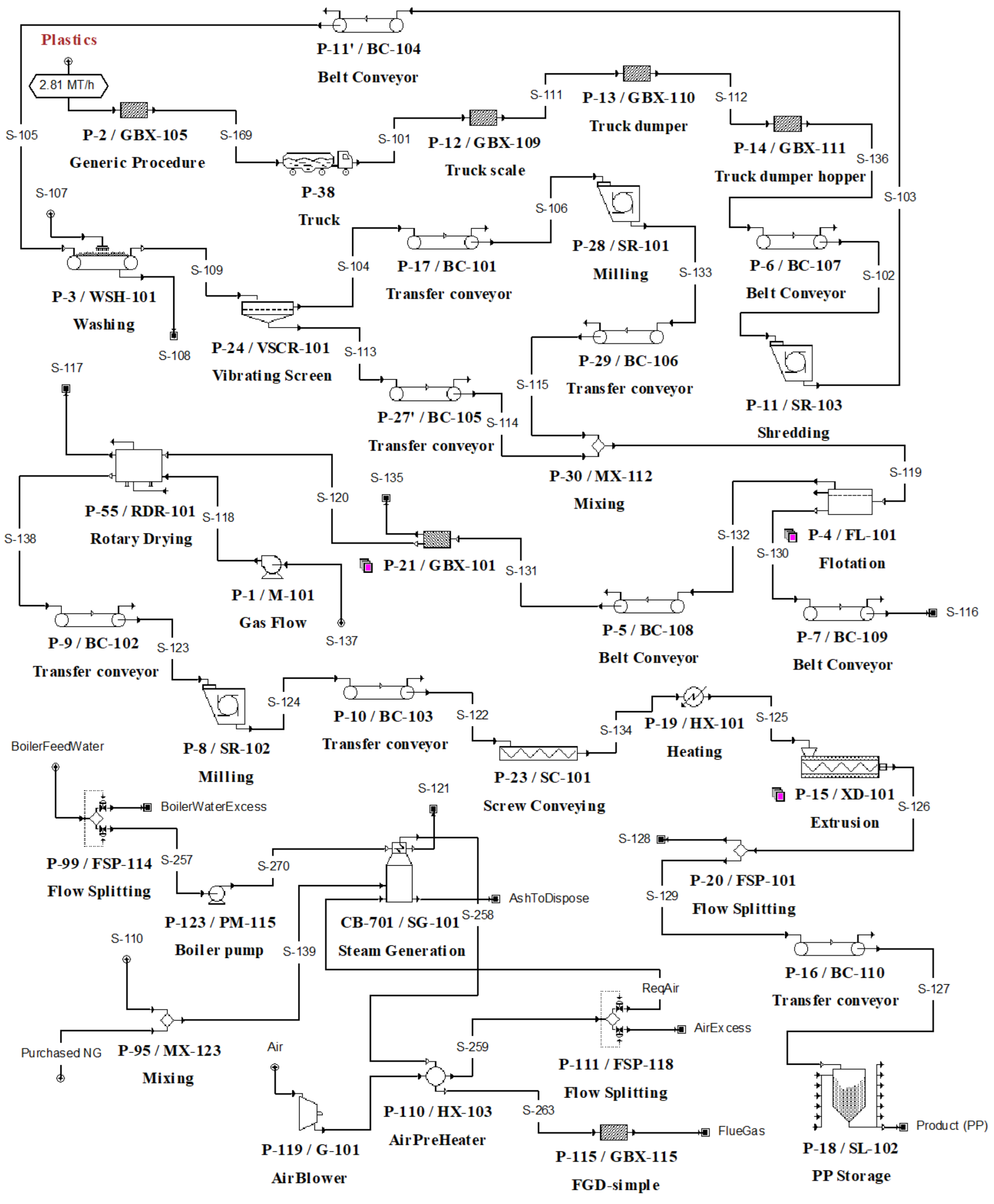


**Fig S3. Overview of process model developed in SuperPro Designer**

*Energy Consumption Results*

The energy consumption results, normalized in units of kWh per tonne, are presented in Figure S4 and Table S1 below. Our modeling efforts in SuperPro Designer yield full material and energy balance data for mechanical recycling processes. These results are only used in the LCA of solvent-assisted recycling to model pretreatment to PP dissolution; they are not used in the LCA of PP mechanical recycling because real-life facility-scale data is available in literature (12).

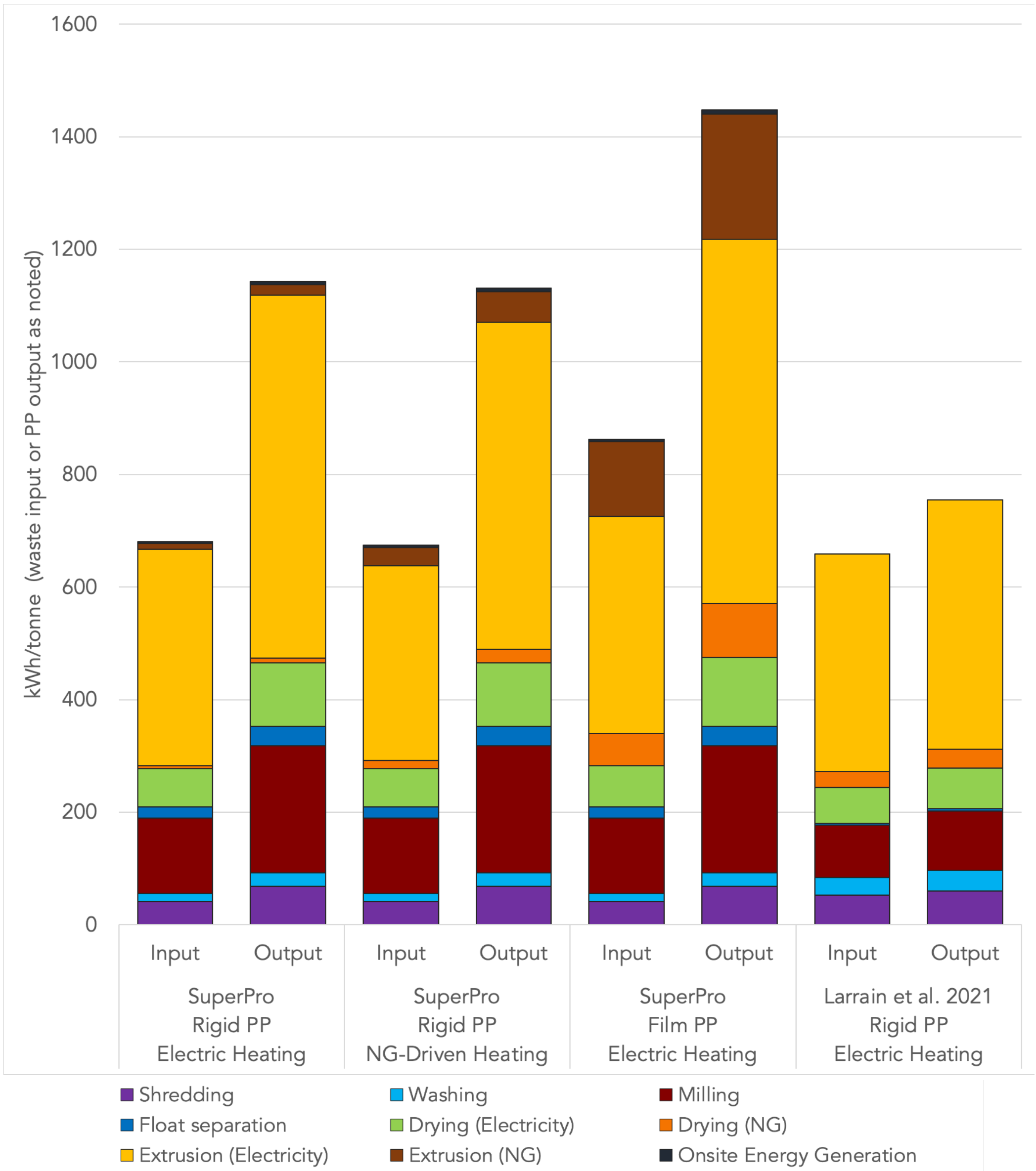


**Figure S4. Mechanical Recycling Energy Consumption by Unit Process**

## Table S1. Mechanical Recycling Energy Consumption by Unit Process (Tabulated Data for Fig. S4)

| Model | Feedstock | Heating Scenario | Shredding | Washing | Milling | Float separation | Drying (Electricity) | Drying (NG) | Extrusion (Electricity) | Extrusion (NG) | Onsite Energy Generation | Total | Units |
|---|---|---|---|---|---|---|---|---|---|---|---|---|---|
| SuperPro | Rigid PP | Electric Heating | 41 | 15 | 134 | 20 | 68 | 5 | 385 | 11 | 3 | 681 | kWh/tonne input |
| | | | 68 | 25 | 225 | 34 | 114 | 8 | 645 | 18 | 6 | 1142 | kWh/tonne output |
| SuperPro | Rigid PP | NG-Driven Heating | 41 | 15 | 134 | 20 | 68 | 14 | 346 | 33 | 3 | 674 | kWh/tonne input |
| | | | 68 | 25 | 225 | 34 | 114 | 24 | 581 | 55 | 6 | 1131 | kWh/tonne output |
| SuperPro | Film PP | Electric Heating | 41 | 15 | 134 | 20 | 73 | 57 | 386 | 133 | 4 | 863 | kWh/tonne input |
| | | | 68 | 25 | 225 | 34 | 123 | 96 | 647 | 223 | 7 | 1447 | kWh/tonne output |
| Larrain et al. 2021 | Rigid PP | Electric Heating | 53 | 32 | 92 | 4 | 63 | 28 | 387 | 0 | 0 | 659 | kWh/tonne input |
| | | | 60 | 36 | 106 | 4 | 72 | 33 | 443 | 0 | 0 | 754 | kWh/tonne output |

## 4. Life Cycle Assessment

*LCA Scenarios*

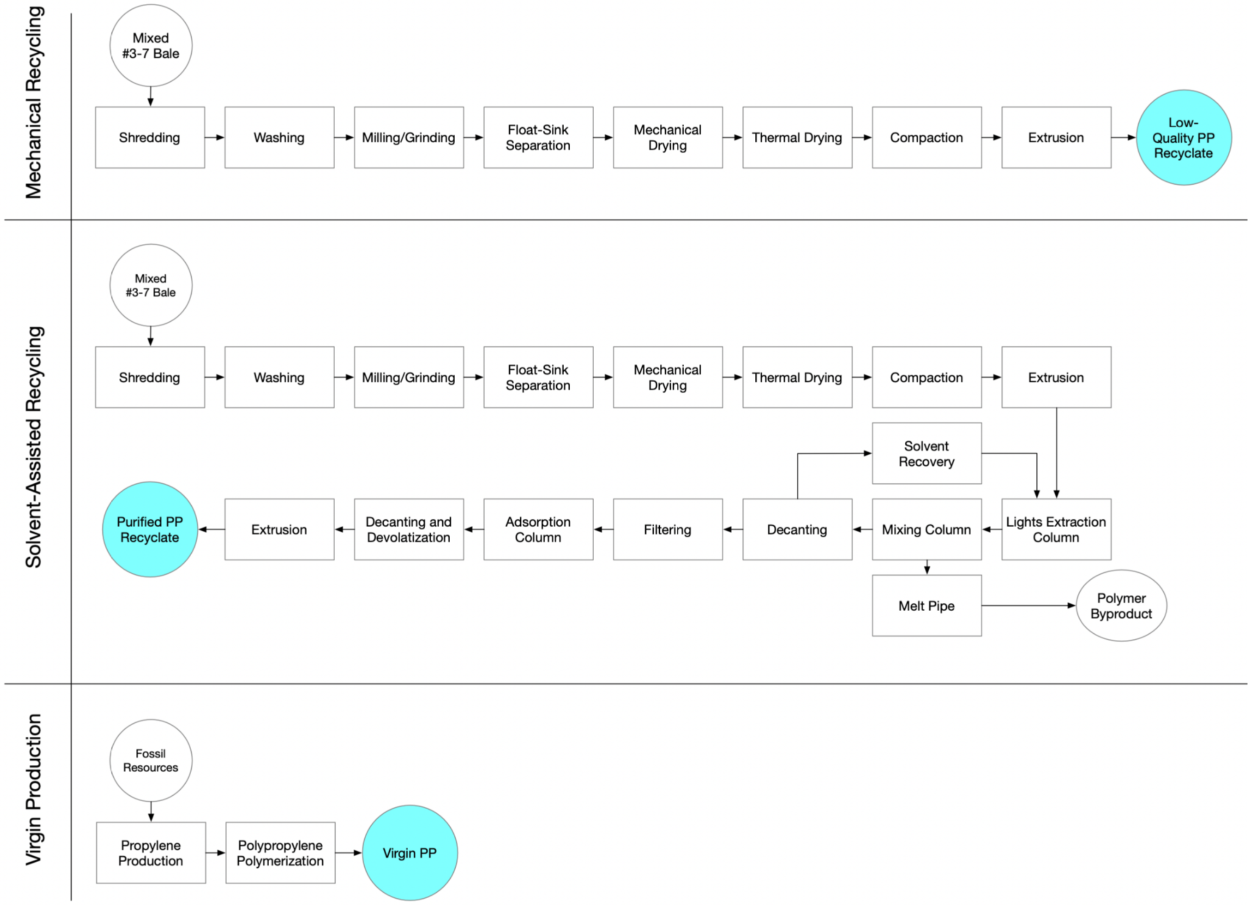


**Figure S5. LCA Scenario Processes**

**Table S2. Life-Cycle Inventory (LCI) Descriptions by Scenario**

| Scenario | Type of Direct Requirements |
|---|---|
| Mechanical Recycling (main data source: (12)) | Transportation (trucking and rail),<br>Energy (electricity and natural gas),<br>Washing agent (NaOH) |
| Solvent-Assisted Recycling (main data source is confidential, but reflects pilot-scale operations in the U.S.) | Transportation (trucking and rail),<br>Energy (electricity and natural gas),<br>Solvent (butane),<br>Other process materials (NaOH, silica, alumina),<br>Antioxidant additives (assume pentaerythritol and irgafos 168 as proxies),<br>Antistatic additives (assume stearic acid as proxy), |

| | |
|---|---|
| | Crude oil offset (polymer byproduct stream is assumed to offset crude oil production based on energy content) |
| Virgin Production<br>(main data source: (2)) | Raw material extraction (crude oil and natural gas),<br>Energy (electricity, natural gas, other petroleum-based fuels),<br>Transportation (trucking and barge) |

*LCA Model: Calculations*

The basic math upon which the LCA model is built is provided below:

$$(I - A)X = Y$$

N = total number of unit processes
$A$ = NxN input-output matrix with life-cycle inventories for each unit process (non-zero data listed in Table S4)
$I$ = NxN identity matrix
$Y$ = N length vector w/ direct requirements for scenario analysis
$X$ = N length vector w/ life-cycle requirements for scenario analysis
$E$ = N length vector w/ emission factors for each unit process (emission factors by pollutant type are provided in Table S3)

$$X = [x_i \vdots x_N] \qquad E = [e_i \vdots e_N]$$

$$LifeCycle\ Emissions = \sum_{i}^{N} x_i * e_i$$

*LCA Model: Data*

**Table S3. LCA Model: Emission Factors**

All values are in units of kg of pollutant (given by column name) per unit indicated in the unit process name. These are not necessarily life-cycle emission factors and only definitely include direct emission impacts (in several cases, this means fugitive emissions associated with fuel combustion); full life-cycle impacts must be assessed through the model using this data along with IO data from Table S4. When possible, GHG emission factors are separated by pollutant type; in cases where this is not possible, total GHG impact in $CO_2$ equivalence is given by the $CO_2$ column while the $CH_4$ and $N_2O$ columns are marked with zeros.

| Unit Process | $CO_2$ | $CH_4$ | $N_2O$ | Source |
|---|---|---|---|---|
| alumina.kg | 1.93E+00 | 3.54E-03 | 4.70E-05 | GREET 2020 (38) |
| barge.mt_km | 2.22E-02 | 0.00E+00 | 0.00E+00 | Cohon et al. 2010 (39) |
| butane.MJ | 5.16E-03 | 2.24E-07 | 4.35E-08 | GREET 2021 (40) |
| caco3.kg | 1.61E-03 | 5.15E-08 | 3.19E-08 | GREET 2020 (38) |
| coal.MJ | 6.97E-04 | 1.39E-04 | 8.73E-09 | GREET 2018 (41) |
| crudeoil.MJ | 2.62E-03 | 8.24E-05 | 3.12E-08 | GREET 2019 (42) |

| | | | | |
|---|---|---|---|---|
| diesel.MJ | 5.09E-03 | 3.93E-08 | 2.02E-07 | GREET 2020 (38) |
| electricity.MRO.kWh | 6.03E-01 | 7.54E-05 | 1.09E-05 | Ou and Cai 2020 (43) |
| electricity.NGCC.kWh | 4.05E-01 | 7.46E-06 | 7.36E-07 | Ou and Cai 2020 (43) |
| electricity.nuclear.kWh | 0.00E+00 | 0.00E+00 | 0.00E+00 | NA |
| electricity.TRE.kWh | 4.20E-01 | 3.60E-05 | 5.00E-06 | Ou and Cai 2020 (43) |
| electricity.US.kWh | 4.05E-01 | 7.00E-06 | 4.00E-06 | Ou and Cai 2020 (43) |
| electricity.WECC.kWh | 3.27E-01 | 3.41E-05 | 5.03E-06 | Ou and Cai 2020 (43) |
| flatbedtruck.mt_km | 1.24E-01 | 0.00E+00 | 0.00E+00 | Cohon et al. 2010 (39) |
| formaldehyde.kg | 0.00E+00 | 0.00E+00 | 0.00E+00 | GREET 2021 (40) |
| gasoline.MJ | 9.23E-03 | 3.12E-06 | 8.64E-08 | Lu et al. 2016 (44) |
| gaspipeline.mt_km | 0.00E+00 | 0.00E+00 | 0.00E+00 | NA |
| glycerin.kg | 1.91E-01 | 4.90E-04 | 2.69E-06 | GREET 2018 (41) |
| h2.kg | 1.06E+01 | 5.98E-02 | 4.00E-05 | Spath and Mann 2001 (45) |
| irgafos168.kg | 4.68E-01 | 0.00E+00 | 0.00E+00 | Assumed tris(2,4-ditert-butylphenyl) phosphite as proxy; Ecoinvent v3.9 (46) |
| landfill_inorganics_wet.kg | 0.00E+00 | 0.00E+00 | 0.00E+00 | WARM v14 (47) |
| landfill_mixedMSW.wet_kg | 0.00E+00 | 2.87E-02 | 0.00E+00 | WARM v14 (47) |
| landfill_mixedorganics.wet_kg | 0.00E+00 | 2.18E-02 | 0.00E+00 | WARM v14 (47) |
| lime.kg | 1.11E+00 | 9.54E-07 | 9.86E-08 | GREET 2020 (38) |
| liquidpipeline.mt_km | 0.00E+00 | 0.00E+00 | 0.00E+00 | NA |
| lpg.kg | 5.22E-01 | 1.45E-03 | 8.28E-06 | GREET 2021 (40) |
| marinetanker.mt_km | 6.91E-03 | 8.02E-08 | 0.00E+00 | Cohon et al. 2010 (39) |
| methanol.kg | 4.05E-01 | 4.67E-03 | 7.19E-06 | GREET 2021 (40) |
| na_brine.kg | 5.47E-02 | 1.19E-06 | 6.20E-07 | GREET 2020 (38) |
| naoh.kg | 4.68E-01 | 8.09E-06 | 4.32E-06 | GREET 2020 (38) |
| naturalgas_combust.MJ | 5.04E-02 | 0.00E+00 | 0.00E+00 | Based on stoichiometry of methane combustion; Assumes perfect oxidation |
| naturalgas_select.MJ | 0.00E+00 | 0.00E+00 | 0.00E+00 | NA |
| naturalgas.conventional.MJ | 4.06E-03 | 8.70E-05 | 2.60E-08 | GREET 2020 (38) |
| naturalgas.shale.MJ | 3.80E-03 | 9.19E-05 | 2.59E-08 | GREET 2020 (38) |
| o2.kg | 0.00E+00 | 0.00E+00 | 0.00E+00 | NA |
| pentaerythritol.kg | 3.31E-01 | 0.00E+00 | 0.00E+00 | Ecoinvent v3.9 (46) |
| rail.mt_km | 1.86E-02 | 0.00E+00 | 0.00E+00 | Cohon et al. 2010 (39) |
| refgas.MJ | 0.00E+00 | 0.00E+00 | 0.00E+00 | NA |
| rfo.MJ | 4.00E-03 | 8.74E-06 | 6.83E-08 | GREET 2019 (42) |
| silica.kg | 0.00E+00 | 0.00E+00 | 0.00E+00 | GREET 2022 (48) |
| stearic_acid.kg | 1.08E-01 | 0.00E+00 | 0.00E+00 | Ecoinvent v3.9 (46) |
| tankertruck.mt_km | 8.46E-02 | 0.00E+00 | 0.00E+00 | Cohon et al. 2010 (39) |
| uranium.kg | 1.12E+01 | 0.00E+00 | 0.00E+00 | Parker et al. 2016 (49) |

| | | | | |
|---|---|---|---|---|
| LPG_combust_industrialboiler.MJ | 6.45E-02 | 1.01E-06 | 4.56E-06 | GREET 2020 (38) |
| diesel_combust_industrialboiler.MJ | 7.41E-02 | 1.88E-07 | 8.70E-07 | GREET 2020 (38) |
| residualoil_combust_industrialboiler.MJ | 8.06E-02 | 3.06E-06 | 1.62E-06 | GREET 2020 (38) |
| gasoline_combust_industrialboiler.MJ | 6.88E-02 | 2.84E-06 | 5.69E-07 | GREET 2020 (38) |
| offgas_combust_industrialboiler.MJ | 5.77E-02 | 3.04E-06 | 5.88E-07 | GREET 2020 (38) |

**Table S4. LCA Model: Input-Output Matrix Relationships**

Our LCA model uses a physical units-based input-output matrix that is populated with life-cycle inventories for each unit process/product included. The relevant non-zero values are included in this table. Each unit process/product is listed with a unit. The value indicates the amount of the upstream/downstream requirement in its listed unit required to make 1 unit of the primary unit product/process. If any unit processes from Table S3 is not included in this table, there are no appreciable upstream/downstream impacts for that parameter.

| Unit Process | Upstream/Downstream Requirements | Value** | Source |
|---|---|---|---|
| electricity.US.kWh | | | |
| | coal.MJ | 2.59E+00 | Ou and Cai 2020 (43) |
| | diesel.MJ | 6.75E-03 | Ou and Cai 2020 (43) |
| | rfo.MJ | 4.51E-02 | Ou and Cai 2020 (43) |
| | electricity.US.kWh | 4.90E-02 | Ou and Cai 2020 (43) |
| | naturalgas_select.MJ | 2.77E+00 | Ou and Cai 2020 (43) |
| | electricity.nuclear.kWh | 2.15E-01 | Ou and Cai 2020 (43) |
| flatbedtruck.mt_km | | | |
| | diesel.MJ | 1.78E+00 | Cohon et al. 2010 (39) |
| | flatbedtruck.mt_km | 2.50E-01 | Cohon et al. 2010 (39) |
| rail.mt_km | | | Cohon et al. 2010 (39) |
| | diesel.MJ | 2.68E-01 | Cohon et al. 2010 (39) |
| | rail.mt_km | 2.50E-01 | Cohon et al. 2010 (39) |
| naturalgas_select.MJ | | | |
| | naturalgas.conventional.MJ | 8.35E-01 | Burnham 2018 (50) |
| | naturalgas.shale.MJ | 1.65E-01 | Burnham 2018 (50) |
| diesel.MJ | | | |
| | rfo.MJ | 3.11E-02 | GREET 2020 (38) |
| | refgas.MJ | 5.81E-02 | GREET 2020 (38) |
| | crudeoil.MJ | 1.00E+00 | GREET 2020 (38) |
| | electricity.US.kWh | 8.93E-04 | GREET 2020 (38) |
| | h2.kg | 1.08E-04 | GREET 2020 (38) |
| | naturalgas_select.MJ | 5.19E-02 | GREET 2020 (38) |

| | | | |
|---|---|---|---|
| | tankertruck.mt_km | 3.53E-03 | GREET 2020 (38) |
| | liquidpipeline.mt_km | 2.10E-02 | GREET 2020 (38) |
| | butane.MJ | 9.92E-05 | GREET 2020 (38) |
| naoh.kg | | | |
| | coal.MJ | 6.01E-01 | GREET 2020 (38) |
| | rfo.MJ | 2.11E-02 | GREET 2020 (38) |
| | electricity.US.kWh | 1.67E+00 | GREET 2020 (38) |
| | naturalgas_select.MJ | 7.28E+00 | GREET 2020 (38) |
| | tankertruck.mt_km | 4.26E-01 | GREET 2020 (38) |
| | rail.mt_km | 1.26E+00 | GREET 2020 (38) |
| | na_brine.kg | 5.83E+00 | GREET 2020 (38) |
| butane.MJ | | | |
| | naturalgas_select.MJ | 1.00E+00 | NA |
| crudeoil.MJ | | | |
| | diesel.MJ | 3.06E-03 | GREET 2018 (41) |
| | rfo.MJ | 2.04E-04 | GREET 2018 (41) |
| | crudeoil.MJ | 2.04E-04 | GREET 2018 (41) |
| | electricity.US.kWh | 1.08E-03 | GREET 2018 (41) |
| | gasoline.MJ | 4.08E-04 | GREET 2018 (41) |
| | naturalgas_select.MJ | 1.26E-02 | GREET 2018 (41) |
| | tankertruck.mt_km | 5.49E-03 | GREET 2018 (41) |
| | liquidpipeline.mt_km | 2.85E-02 | GREET 2018 (41) |
| | rail.mt_km | 2.42E-02 | GREET 2018 (41) |
| | barge.mt_km | 4.39E-03 | GREET 2018 (41) |
| | marinetanker.mt_km | 1.10E-01 | GREET 2018 (41) |
| silica.kg | | | |
| | electricity.US.kWh | 5.17E-05 | GREET 2022 (48) |
| | flatbedtruck.mt_km | 8.00E-02 | General assumption (50 mi by truck) |
| | rail.mt_km | 8.00E-01 | General assumption (500 mi by rail) |
| pentaerythritol.kg | | | |
| | electricity.US.kWh | 4.16E-01 | Ecoinvent v3.9 (46) |
| | naturalgas_select.MJ | 4.00E+00 | Ecoinvent v3.9 (46) |
| | formaldehyde.kg | 1.04E+00 | Ecoinvent v3.9 (46) |
| irgafos168.kg | | | |
| | electricity.US.kWh | 1.25E+00 | Assumed tris(2,4-ditert-butylphenyl) phosphite as proxy; Ecoinvent v3.9 (46) |
| | naturalgas_select.MJ | 6.45E+00 | Assumed tris(2,4-ditert-butylphenyl) phosphite as proxy; |

| | | | |
|---|---|---|---|
| | | | Ecoinvent v3.9 (46) |
| stearic_acid.kg | | | |
| | glycerin.kg | -1.08E-01 | Ecoinvent v3.9 (46) |
| | electricity.US.kWh | 4.16E-01 | Ecoinvent v3.9 (46) |
| | naturalgas_select.MJ | 2.15E+00 | Ecoinvent v3.9 (46) |
| electricity.TRE.kWh | | | |
| | coal.MJ | 2.04E+00 | Ou and Cai 2020 (43) |
| | diesel.MJ | 1.91E-04 | Ou and Cai 2020 (43) |
| | rfo.MJ | 9.01E-03 | Ou and Cai 2020 (43) |
| | electricity.TRE.kWh | 4.90E-02 | Ou and Cai 2020 (43) |
| | naturalgas_select.MJ | 4.03E+00 | Ou and Cai 2020 (43) |
| | electricity.nuclear.kWh | 1.13E-01 | Ou and Cai 2020 (43) |
| rfo.MJ | | | |
| | rfo.MJ | 2.70E-02 | GREET 2019 (42) |
| | refgas.MJ | 3.73E-02 | GREET 2019 (42) |
| | crudeoil.MJ | 1.00E+00 | GREET 2019 (42) |
| | electricity.US.kWh | 3.71E-04 | GREET 2019 (42) |
| | h2.kg | 1.20E-05 | GREET 2019 (42) |
| | naturalgas_select.MJ | 2.43E-02 | GREET 2019 (42) |
| | tankertruck.mt_km | 3.53E-03 | GREET 2019 (42) |
| | liquidpipeline.mt_km | 2.10E-02 | GREET 2019 (42) |
| | butane.MJ | 7.20E-05 | GREET 2019 (42) |
| gasoline.MJ | | | |
| | rfo.MJ | 9.28E-02 | Lu et al. 2016 (44) |
| | refgas.MJ | 9.26E-02 | Lu et al. 2016 (44) |
| | crudeoil.MJ | 1.00E+00 | Lu et al. 2016 (44) |
| | electricity.US.kWh | 1.44E-02 | Lu et al. 2016 (44) |
| | h2.kg | 5.26E-05 | Lu et al. 2016 (44) |
| | naturalgas_select.MJ | 6.27E-02 | Lu et al. 2016 (44) |
| | tankertruck.mt_km | 3.53E-03 | Lu et al. 2016 (44) |
| | liquidpipeline.mt_km | 2.10E-02 | Lu et al. 2016 (44) |
| | butane.MJ | 6.44E-02 | Lu et al. 2016 (44) |
| barge.mt_km | | | |
| | diesel.MJ | 3.20E-01 | Cohon et al. 2010 (39) |
| | barge.mt_km | 2.50E-01 | Cohon et al. 2010 (39) |
| liquidpipeline.mt_km | | | |
| | electricity.US.kWh | 1.84E-02 | Scown et al. 2012 (51) |
| coal.MJ | | | |
| | diesel.MJ | 2.30E-03 | GREET 2018 (41) |
| | rfo.MJ | 2.53E-04 | GREET 2018 (41) |

| | | | |
|---|---|---|---|
| | electricity.US.kWh | 2.09E-03 | GREET 2018 (41) |
| | gasoline.MJ | 1.89E-04 | GREET 2018 (41) |
| | naturalgas_select.MJ | 5.82E-05 | GREET 2018 (41) |
| | flatbedtruck.mt_km | 2.55E-03 | GREET 2018 (41) |
| | rail.mt_km | 5.34E-02 | GREET 2018 (41) |
| | barge.mt_km | 2.24E-02 | GREET 2018 (41) |
| electricity.nuclear.kWh | | | |
| | electricity.US.kWh | 1.97E-03 | Warner and Heath 2012 (52) |
| | naturalgas_select.MJ | 1.52E-01 | Warner and Heath 2012 (52) |
| | uranium.kg | 3.83E-08 | Warner and Heath 2012 (52) |
| naturalgas.conventional.MJ | | | |
| | diesel.MJ | 3.09E-03 | Burnham 2018 (50) |
| | rfo.MJ | 2.56E-04 | Burnham 2018 (50) |
| | electricity.NGCC.kWh | 2.98E-04 | Burnham 2018 (50) |
| | gasoline.MJ | 2.56E-04 | Burnham 2018 (50) |
| | naturalgas.conventional.MJ | 4.81E-02 | Burnham 2018 (50) |
| | gaspipeline.mt_km | 7.66E-02 | Burnham 2018 (50) |
| naturalgas.shale.MJ | | | |
| | diesel.MJ | 2.95E-03 | Burnham 2018 (50) |
| | rfo.MJ | 2.44E-04 | Burnham 2018 (50) |
| | electricity.US.kWh | 2.95E-04 | Burnham 2018 (50) |
| | gasoline.MJ | 2.44E-04 | Burnham 2018 (50) |
| | naturalgas.shale.MJ | 4.71E-02 | Burnham 2018 (50) |
| | gaspipeline.mt_km | 7.66E-02 | Burnham 2018 (50) |
| refgas.MJ | | | |
| | crudeoil.MJ | 1.00E+00 | NA |
| h2.kg | | | |
| | electricity.US.kWh | 2.69E-01 | Spath and Mann 2001 (45) |
| | naturalgas_select.MJ | 1.43E+02 | Spath and Mann 2001 (45) |
| | gaspipeline.mt_km | 1.21E+00 | Spath and Mann 2001 (45) |
| tankertruck.mt_km | | | |
| | diesel.MJ | 1.22E+00 | Cohon et al. 2010 (39) |
| | tankertruck.mt_km | 2.50E-01 | Cohon et al. 2010 (39) |
| na_brine.kg | | | |
| | rfo.MJ | 1.28E-01 | GREET 2020 (38) |
| | electricity.US.kWh | 2.56E-01 | GREET 2020 (38) |
| | naturalgas_select.MJ | 7.91E-01 | GREET 2020 (38) |
| marinetanker.mt_km | | | |
| | rfo.MJ | 1.00E-01 | Cohon et al. 2010 (39) |
| | marinetanker.mt_km | 2.50E-01 | Cohon et al. 2010 (39) |

| | | | |
|---|---|---|---|
| formaldehyde.kg | | | |
| | electricity.US.kWh | 1.50E-01 | GREET 2021 (40) |
| | methanol.kg | 1.20E+00 | GREET 2021 (40) |
| methanol.kg | | | |
| | electricity.US.kWh | 8.05E-03 | GREET 2019 (42) |
| | naturalgas_select.MJ | 6.93E+00 | GREET 2019 (42) |
| | tankertruck.mt_km | 9.04E-03 | GREET 2019 (42) |
| | gaspipeline.mt_km | 9.05E-01 | GREET 2019 (42) |
| | o2.kg | 3.80E-01 | GREET 2019 (42) |
| uranium.kg | | | |
| | lime.kg | 2.91E+00 | Parker et al. 2016 (49) |
| | diesel.MJ | 2.39E-09 | Parker et al. 2016 (49) |
| | electricity.WECC.kWh | 2.37E+01 | Parker et al. 2016 (49) |
| | electricity.MRO.kWh | 4.35E+01 | Parker et al. 2016 (49) |
| | gasoline.MJ | 3.30E-11 | Parker et al. 2016 (49) |
| | naturalgas_select.MJ | 1.16E-06 | Parker et al. 2016 (49) |
| | flatbedtruck.mt_km | 1.17E+01 | Parker et al. 2016 (49) |
| | lpg.kg | 2.53E+00 | Parker et al. 2016 (49) |
| | landfill_mixedorganics.wet_kg | 1.68E-01 | Parker et al. 2016 (49) |
| | landfill_mixedMSW.wet_kg | 1.22E-01 | Parker et al. 2016 (49) |
| electricity.NGCC.kWh | | | |
| | electricity.NGCC.kWh | 6.50E-02 | Ou and Cai 2020 (43) |
| | naturalgas_select.MJ | 7.20E+00 | Ou and Cai 2020 (43) |
| electricity.WECC.kWh | | | |
| | coal.MJ | 1.92E+00 | Ou and Cai 2020 (43) |
| | diesel.MJ | 9.37E-04 | Ou and Cai 2020 (43) |
| | rfo.MJ | 1.59E-02 | Ou and Cai 2020 (43) |
| | electricity.WECC.kWh | 4.90E-02 | Ou and Cai 2020 (43) |
| | naturalgas_select.MJ | 2.53E+00 | Ou and Cai 2020 (43) |
| | electricity.nuclear.kWh | 8.83E-02 | Ou and Cai 2020 (43) |
| electricity.MRO.kWh | | | |
| | coal.MJ | 4.80E+00 | Ou and Cai 2020 (43) |
| | diesel.MJ | 3.72E-03 | Ou and Cai 2020 (43) |
| | rfo.MJ | 1.02E-01 | Ou and Cai 2020 (43) |
| | electricity.MRO.kWh | 4.90E-02 | Ou and Cai 2020 (43) |
| | naturalgas_select.MJ | 2.49E+00 | Ou and Cai 2020 (43) |
| | electricity.nuclear.kWh | 1.62E-01 | Ou and Cai 2020 (43) |
| lime.kg | | | |
| | caco3.kg | 1.88E+00 | GREET 2020 (38) |

| | | | |
|---|---|---|---|
| | coal.MJ | 3.57E+00 | GREET 2020 (38) |
| | diesel.MJ | 7.43E-02 | GREET 2020 (38) |
| | rfo.MJ | 3.24E-02 | GREET 2020 (38) |
| | electricity.US.kWh | 5.70E-02 | GREET 2020 (38) |
| | naturalgas_select.MJ | 2.25E-01 | GREET 2020 (38) |
| | tankertruck.mt_km | 1.93E-01 | GREET 2020 (38) |
| | lpg.kg | 4.19E-02 | GREET 2020 (38) |
| caco3.kg | | | |
| | coal.MJ | 3.66E-03 | GREET 2020 (38) |
| | diesel.MJ | 1.30E-02 | GREET 2020 (38) |
| | rfo.MJ | 1.64E-03 | GREET 2020 (38) |
| | electricity.US.kWh | 2.44E-04 | GREET 2020 (38) |
| | gasoline.MJ | 2.56E-03 | GREET 2020 (38) |
| | naturalgas_select.MJ | 1.22E+00 | GREET 2020 (38) |
| | flatbedtruck.mt_km | 8.00E-02 | GREET 2020 (38) |

*Sensitivity Analysis*

To capture uncertainty and variability in process yield, energy consumption, and transportation impacts for the PP recycling scenarios, we conduct 10,000 Monte Carlo simulations using triangular probability distributions as given by Table S5. For all parameters, except for energy consumption for mechanical recycling, the mode is equivalent to the original model value. Because the original energy use data for mechanical recycling is not broken down by pretreatment and extrusion (12), we opted to use the SuperPro Designer results for the modes instead of the original model data. The total energy consumption for mechanical recycling used in the original LCA falls within the aggregated ranges in Table S5.  For mechanical processes, we use specific minimum and maximum values from SuperPro Designer and Larrain et al. (2021) (53). For all other parameter probability distributions, the maximum and minimum values are equally spaced from the mode.

**Table S5. Recycling Scenarios' Sensitivity Analyses: Probability Distributions for Monte Carlo Simulations**

| Scenario | Parameter | Values | | | | Sources | | |
|---|---|---|---|---|---|---|---|---|
| | | mode | max | min | units | mode | max | min |
| Solvent-Assisted Recycling | Pretreatment electricity consumption | 281.0 | 287.0 | 243.5 | kWh/tonne input | SuperPro (rigids, electric extrusion) | SuperPro (films, NG-driven extrusion) | Larrain et al. (2021) (53) |
| | Pretreatment natural gas consumption | 16.4 | 102.4 | 16.4 | MJ/tonne input | SuperPro (electric extrusion) | Larrain et al. (2021) (53) | SuperPro (electric extrusion) |
| | Process yield | x | x + 3 % | x - 3 % | % | Confidential | N/A | N/A |
| | Electricity consumption | x | x + 10 % | x - 10 % | kWh/tonne input | Confidential | N/A | N/A |
| | Natural gas consumption | x | x + 10 % | x - 10 % | MJ/tonne input | Confidential | N/A | N/A |
| | Byproduct stream | x | x + 10 % | x - 10 % | N/A | Confidential | N/A | N/A |
| | Transportation (trucking) | 0.77 | mode + 5 % | mode - 5 % | tonne-km/ kg input | Franklin Associates (2018) (12) | N/A | N/A |
| | Transportation (rail) | 0.29 | mode + 5 % | mode - 5 % | tonne-km/ kg input | Franklin Associates (2018) (12) | N/A | N/A |
| Mechanical Recycling | Pretreatment electricity consumption | 281.0 | 287.0 | 243.5 | kWh/tonne input | SuperPro (electric extrusion) | SuperPro (films, NG-driven extrusion) | Larrain et al. (2021) (53) |
| | Pretreatment natural gas consumption | 16.4 | 102.4 | 16.4 | MJ/tonne input | SuperPro (electric extrusion) | Larrain et al. (2021) (53) | SuperPro (electric extrusion) |
| | Process yield | 85 | 87 | 55 | % | Franklin Associates (2018) (12) | Larrain et al. (2021) (53) | SuperPro (electric extrusion) |
| | Electricity consumption for extrusion | 384.7 | 386.9 | 209.5 | kWh/tonne input | SuperPro (electric extrusion) | Larrain et al. (2021) (53) | Total from Franklin Associates (2018) (12) minus pretreatment from Larrain et al. (2021) (53) |
| | Natural gas consumption for extrusion | 38.3 | 694.4 | 0.0 | MJ/tonne input | SuperPro (electric extrusion) | Total from Franklin Associates (2018) (12) minus pretreatment from Larrain et al. (2021) (53) | Larrain et al. (2021) (53) |
| | Transportation (trucking) | 0.77 | mode + 5 % | mode - 5 % | tonne-km/ kg input | Franklin Associates (2018) (12) | N/A | N/A |
| | Transportation (rail) | 0.29 | mode + 5 % | mode - 5 % | tonne-km/ kg input | Franklin Associates (2018) (12) | N/A | N/A |

**Table S6. Boxplot Data for Virgin PP Production (Figure 1 in main text)**

| Source | GHG emission factor (kg $CO_{2e}$ per tonne PP output) |
|---|---|
| Franklin Associates (12) | 1840 |
| Franklin Associates 2011 (2) | 1860 |
| Franklin Associates 2021 (54) | 1548 |
| Nicholson 2021 (film) (55) | 2500 |
| Nicholson 2021 (IM) (55) | 2700 |

# 5. LCA Results: Tabulated Data

**Table S7. Life-Cycle Greenhouse Gas Impacts from Virgin Production and Recycling (Data for Figure 2)**

| | Mechanical Recycling | Solvent-Assisted Recycling | Virgin Production |
|---|---|---|---|
| Raw Materials | 0.0 | 0.0 | 394.5 |
| Preprocessing* | 1.7 | 139.1 | 0.0 |
| Solvent | 0.0 | 11.7 | 0.0 |
| Process Materials | 0.0 | 103.2 | 0.0 |
| Byproducts | 0.0 | -43.9 | 0.0 |
| Direct Emissions | 0.0 | 0.0 | 253.5 |
| Electricity | 250.8 | 607.4 | 290.0 |
| Natural Gas | 53.5 | 652.3 | 527.1 |
| Non-NG Fuels | 3.8 | 0.0 | 792.4 |

| | | | |
|---|---|---|---|
| Transportation | 168.7 | 160.6 | 91.8 |

*Preprocessing for solvent-assisted recycling includes much of what is considered part of the main process for mechanical recycling. This includes electricity and natural gas use. For mechanical recycling, preprocessing includes washing agents. Units for all values are kg $CO_{2eq}$ per tonne PP produced.

**Table S8. Emission Factors Forecast for PP Production and Recycling Emission (Data for Figure 4)**

| | CA, Mid | | | CA, Low Renewable Cost | | | US Ave, Mid | | | US Ave, Low Renewable Cost | | |
|---|---|---|---|---|---|---|---|---|---|---|---|---|
| Year | Mechanical Recycling | Solvent-Assisted Recycling | Virgin Production | Mechanical Recycling | Solvent-Assisted Recycling | Virgin Production | Mechanical Recycling | Solvent-Assisted Recycling | Virgin Production | Mechanical Recycling | Solvent-Assisted Recycling | Virgin Production |
| 2022 | 354.6 | 1271.0 | 2264.2 | 351.6 | 1262.0 | 2262.0 | 466.2 | 1602.6 | 2345.9 | 465.9 | 1601.8 | 2345.7 |
| 2024 | 331.3 | 1201.7 | 2247.1 | 332.8 | 1206.3 | 2248.3 | 419.8 | 1464.9 | 2311.9 | 410.2 | 1436.2 | 2304.9 |
| 2026 | 318.8 | 1164.5 | 2238.0 | 317.4 | 1160.5 | 2237.0 | 394.0 | 1388.2 | 2293.1 | 374.4 | 1329.7 | 2278.6 |
| 2028 | 296.3 | 1097.8 | 2221.5 | 284.6 | 1062.8 | 2212.9 | 341.9 | 1233.3 | 2254.9 | 301.0 | 1111.8 | 2225.0 |
| 2030 | 269.4 | 1017.7 | 2201.8 | 256.3 | 978.8 | 2192.2 | 311.3 | 1142.2 | 2232.5 | 278.3 | 1044.2 | 2208.3 |
| 2032 | 263.3 | 999.6 | 2197.4 | 252.4 | 967.1 | 2189.4 | 302.4 | 1115.9 | 2226.0 | 270.6 | 1021.3 | 2202.7 |
| 2034 | 258.8 | 986.3 | 2194.1 | 251.3 | 963.9 | 2188.6 | 291.9 | 1084.5 | 2218.3 | 264.1 | 1002.0 | 2197.9 |
| 2036 | 255.4 | 976.1 | 2191.6 | 249.5 | 958.7 | 2187.3 | 286.0 | 1067.1 | 2214.0 | 259.7 | 988.8 | 2194.7 |
| 2038 | 251.7 | 965.2 | 2188.9 | 248.5 | 955.7 | 2186.5 | 282.2 | 1055.8 | 2211.2 | 260.2 | 990.2 | 2195.0 |
| 2040 | 249.1 | 957.3 | 2186.9 | 247.8 | 953.4 | 2186.0 | 278.6 | 1045.1 | 2208.6 | 266.4 | 1008.9 | 2199.6 |
| 2042 | 247.2 | 951.6 | 2185.5 | 247.3 | 952.0 | 2185.6 | 276.9 | 1040.0 | 2207.3 | 271.9 | 1025.1 | 2203.6 |
| 2044 | 246.0 | 948.2 | 2184.7 | 246.5 | 949.8 | 2185.1 | 277.4 | 1041.6 | 2207.7 | 275.6 | 1036.1 | 2206.3 |
| 2046 | 245.5 | 946.7 | 2184.3 | 245.9 | 947.9 | 2184.6 | 282.5 | 1056.6 | 2211.4 | 270.3 | 1020.3 | 2202.4 |
| 2048 | 245.1 | 945.6 | 2184.0 | 246.2 | 948.9 | 2184.9 | 288.4 | 1074.1 | 2215.7 | 259.5 | 988.2 | 2194.5 |
| 2050 | 244.7 | 944.3 | 2183.7 | 243.2 | 939.8 | 2182.6 | 294.6 | 1092.6 | 2220.2 | 249.6 | 959.0 | 2187.3 |

The units for all values are kg $CO_{2eq}$ per tonne of PP output.

## References


1. W. Posch, "Polyolefins" in *Applied Plastics Engineering Handbook*, M. Kutz, Ed. (Elsevier, 2011), pp. 23–48.
2. Franklin Associates, "Cradle-to-grave life cycle inventory of nine plastic resins and four polyurethane precursors" (Plastics Division of the American Chemistry Council, 2011).
3. M. J. H. Khan, M. A. Hussain, I. M. Mujtaba, Polypropylene production optimization in fluidized bed catalytic reactor (FBCR): statistical modeling and pilot scale experimental validation. *Materials (Basel)* **7**, 2440–2458 (2014).
4. P. N. Pressley, J. W. Levis, A. Damgaard, M. A. Barlaz, J. F. DeCarolis, Analysis of material recovery facilities for use in life-cycle assessment. *Waste Manag.* **35**, 307–317 (2015).
5. G.A.A. Inc., "2016-2017  Database on material recovery facilities and mixed waste processing facilities in the U.S., with updates" (Governmental Advisory Associates, Inc., 2019).

6. J. Ma, *et al.*, Economic evaluation of infrastructures for thermochemical upcycling of post-consumer plastic waste. *Green Chem.* (2022) https:/doi.org/10.1039/D2GC04005K.

7. T. Hundertmark, M. Prieto, A. Ryba, T. J. Simons, J. Wallach, "Accelerating plastic recovery in the United States" (McKinsey & Company, 2019).

8. R. L. Smith, S. Takkellapati, R. C. Riegerix, Recycling of plastics in the United States: plastic material flows and polyethylene terephthalate (PET) recycling processes. *ACS Sustain. Chem. Eng.* **10**, 2084–2096 (2022).

9. APR, Recycling Rigid Plastics Beyond Bottles: Non-Bottle Containers (2017).

10. Moore Recycling Associates Inc, "National Mixed Rigid PlasticBale Composition Study" (The Association of Plastic Recyclers, 2015).

11. APR, "Model Bale Specifications: 3-7 Bottles and ALL Other Rigid Plastics" (The Association of Plastic Recyclers) (December 7, 2022).

12. Franklin Associates, "Life cycle impacts for postconsumer recycled resins: PET, HDPE and PP" (The Association of Plastic Recyclers, 2018).

13. Z. O. G. Schyns, M. P. Shaver, Mechanical recycling of packaging plastics: A review. *Macromol. Rapid Commun.* **42**, e2000415 (2021).

14. P. Oblak, J. Gonzalez-Gutierrez, B. Zupančič, A. Aulova, I. Emri, Processability and mechanical properties of extensively recycled high density polyethylene. *Polym. Degrad. Stab.* **114**, 133–145 (2015).

15. L. Gustavo Barbosa, M. Piaia, G. Henrique Ceni, Analysis of impact and tensile properties of recycled polypropylene. *IJME* **7**, 117–120 (2017).

16. H. M. da Costa, V. D. Ramos, M. G. de Oliveira, Degradation of polypropylene (PP) during multiple extrusions: Thermal analysis, mechanical properties and analysis of variance. *Polym. Test.* **26**, 676–684 (2007).

17. F. De Santis, R. Pantani, Optical properties of polypropylene upon recycling. *ScientificWorldJournal* **2013**, 354093 (2013).

18. B. Luijsterburg, H. Goossens, Assessment of plastic packaging waste: Material origin, methods, properties. *Resources, Conservation and Recycling* **85**, 88–97 (2014).

19. N. Vidakis, *et al.*, Sustainable additive manufacturing: mechanical response of polypropylene over multiple recycling processes. *Sustainability* **13**, 159 (2020).

20. K. Ragaert, L. Delva, K. Van Geem, Mechanical and chemical recycling of solid plastic waste. *Waste Manag.* **69**, 24–58 (2017).

21. F. Gu, P. Hall, N. J. Miles, Q. Ding, T. Wu, Improvement of mechanical properties of recycled plastic blends via optimizing processing parameters using the Taguchi method and principal component analysis. *Materials & Design (1980-2015)* **62**, 189–198 (2014).

22. A. Ladhari, E. Kucukpinar, H. Stoll, S. Sängerlaub, Comparison of properties with relevance for the automotive sector in mechanically recycled and virgin polypropylene. *Recycling* **6**, 76 (2021).

23. F. Gu, J. Guo, W. Zhang, P. A. Summers, P. Hall, From waste plastics to industrial raw materials: A life cycle assessment of mechanical plastic recycling practice based on a real-world case study. *Sci. Total Environ.* **601–602**, 1192–1207 (2017).

24. E. De Tandt, *et al.*, A recycler's perspective on the implications of REACH and food contact material (FCM) regulations for the mechanical recycling of FCM plastics. *Waste Manag.* **119**, 315–329 (2021).

25. M. K. Eriksen, J. D. Christiansen, A. E. Daugaard, T. F. Astrup, Closing the loop for PET, PE and PP waste from households: Influence of material properties and product design for plastic recycling. *Waste Manag.* **96**, 75–85 (2019).

26. R. R. Bora, R. Wang, F. You, Waste polypropylene plastic recycling toward climate change mitigation and circular economy: energy, environmental, and technoeconomic perspectives. *ACS Sustain. Chem. Eng.* **8**, 16350–16363 (2020).

27. T. Astrup, T. Fruergaard, T. H. Christensen, Recycling of plastic: accounting of greenhouse gases and global warming contributions. *Waste Manag. Res.* **27**, 763–772 (2009).

28. D. Lazarevic, E. Aoustin, N. Buclet, N. Brandt, Plastic waste management in the context of a European recycling society: Comparing results and uncertainties in a life cycle perspective. *Resources, Conservation and Recycling* **55**, 246–259 (2010).

29. M. U. Hossain, S. T. Ng, Y. Dong, B. Amor, Strategies for mitigating plastic wastes management problem: A life cycle assessment study in Hong Kong. *Waste Manag.* **131**, 412–422 (2021).

30. E. Van Eygen, D. Laner, J. Fellner, Integrating high-resolution material flow data into the environmental assessment of waste management system scenarios: the case of plastic packaging in Austria. *Environ. Sci. Technol.* **52**, 10934–10945 (2018).

31. J. Beigbeder, L. Soccalingame, D. Perrin, J.-C. Bénézet, A. Bergeret, How to manage biocomposites wastes end of life? A life cycle assessment approach (LCA) focused on polypropylene (PP)/wood flour and polylactic acid (PLA)/flax fibres biocomposites. *Waste Manag.* **83**, 184–193 (2019).

32. I. T. Mercante, M. D. Bovea, V. Ibáñez-Forés, A. P. Arena, Life cycle assessment of construction and demolition waste management systems: a Spanish case study. *Int. J. Life Cycle Assess.* **17**, 232–241 (2012).

33. G. Faraca, V. Martinez-Sanchez, T. F. Astrup, Environmental life cycle cost assessment: Recycling of hard plastic waste collected at Danish recycling centres. *Resources, Conservation and Recycling* **143**, 299–309 (2019).

34. C. Vadenbo, S. Hellweg, T. F. Astrup, Let's be clear(er) about substitution: a reporting framework to account for product displacement in life cycle assessment. *Journal of Industrial Ecology* **21**, 1078–1089 (2017).

35. L. Rigamonti, M. Grosso, M. C. Sunseri, Influence of assumptions about selection and recycling efficiencies on the LCA of integrated waste management systems. *Int. J. Life Cycle Assess.* **14**, 411–419 (2009).

36. E. Smith, M. M. Bilec, V. Khanna, Evaluating the global plastic waste management system with markov chain material flow analysis. *ACS Sustain. Chem. Eng.* (2023) https:/doi.org/10.1021/acssuschemeng.2c04270.

37. S. Viau, *et al.*, Substitution modelling in life cycle assessment of municipal solid waste management. *Waste Manag.* **102**, 795–803 (2020).

38. M. Wang, *et al.*, Greenhouse gases, Regulated Emissions, and Energy use in Technologies Model ® (2020 Excel). *Argonne National Laboratory (ANL), Argonne, IL (United States)* (2020) https:/doi.org/10.11578/greet-excel-2020/dc.20200912.1.

39. J. L. Cohon, *et al.*, *Hidden costs of energy: unpriced consequences of energy production and use* (National Academies Press, 2010) https:/doi.org/10.17226/12794.

40. M. Wang, *et al.*, Greenhouse gases, Regulated Emissions, and Energy use in Technologies Model ® (2021 Excel). *Argonne National Laboratory (ANL), Argonne, IL (United States)* (2021) https:/doi.org/10.11578/greet-excel-2021/dc.20210902.1.

41. M. Wang, *et al.*, Greenhouse gases, Regulated Emissions, and Energy use in Transportation Model ® (2018 Excel). *Argonne National Laboratory (ANL), Argonne, IL (United States)* (2018) https:/doi.org/10.11578/greet-excel-2018/dc.20200803.1.

42. M. Wang, *et al.*, Greenhouse gases, Regulated Emissions, and Energy use in Transportation Model ® (2019 Excel). *Argonne National Laboratory (ANL), Argonne, IL (United States)* (2019) https:/doi.org/10.11578/greet-excel-2019/dc.20200706.1.

43. L. Ou, H. Cai, "Update of emission factors of greenhouse gases and criteria air pollutants, and generation efficiencies of the U.S. electricity generation sector" (Argonne National Laboratory (ANL), 2020) https:/doi.org/10.2172/1660468.

44. Z. Lu, *et al.*, Well-to-Wheels Analysis of the Greenhouse Gas Emissions and Energy Use of Vehicles with Gasoline Compression Ignition Engines on Low Octane Gasoline-Like Fuel. *SAE Int. J. Fuels Lubr.* **9**, 527–545 (2016).

45. P. Spath, M. Mann, "Life Cycle Assessment of Hydrogen Production via Natural Gas Steam Reforming" (National Renewable Energy Laboratory, 2001).

46. G. Wernet, *et al.*, The ecoinvent database version 3 (part I): overview and methodology. *Int. J. Life Cycle Assess.* **21**, 1218–1230 (2016).

47. EPA, "Documentation for Greenhouse Gas Emission and Energy Factors Used in the

Waste Reduction Model (WARM v.14)" (Office of Resource Conservation and Recovery, U.S. Environmental Protection Agency, 2018).

48. M. Wang, *et al.*, Greenhouse gases, Regulated Emissions, and Energy use in Technologies Model ® (2022 Excel). *Argonne National Laboratory (ANL), Argonne, IL (United States)* (2022) https:/doi.org/10.11578/greet-excel-2022/dc.20220908.1.

49. D. J. Parker, C. S. McNaughton, G. A. Sparks, Life Cycle Greenhouse Gas Emissions from Uranium Mining and Milling in Canada. *Environ. Sci. Technol.* **50**, 9746–9753 (2016).

50. A. Burnham, "Updated Natural Gas Pathways in the GREET1_2018 Model" (Argonne National Laboratory, 2018).

51. C. D. Scown, *et al.*, Lifecycle greenhouse gas implications of US national scenarios for cellulosic ethanol production. *Environmental Research Letters* **7**, 014011 (2012).

52. E. S. Warner, G. A. Heath, Life Cycle Greenhouse Gas Emissions of Nuclear Electricity Generation. *J. Ind. Ecol.* **16**, S73–S92 (2012).

53. M. Larrain, *et al.*, Techno-economic assessment of mechanical recycling of challenging post-consumer plastic packaging waste. *Resources, Conservation and Recycling* **170**, 105607 (2021).

54. Franklin Associates, "Cradle-to-gate life cycle analysis of polypropylene (PP) resin" (American Chemistry Council, 2021).

55. S. R. Nicholson, N. A. Rorrer, A. C. Carpenter, G. T. Beckham, Manufacturing energy and greenhouse gas emissions associated with plastics consumption. *Joule* (2021) https:/doi.org/10.1016/j.joule.2020.12.027.
